# Generalizing Conditional Average Treatment Effects From Nested Randomized Trials to Trial‐Eligible Individuals

**DOI:** 10.1002/sim.70767

**Published:** 2026-10-09

**Authors:** Lan Wen, Issa J. Dahabreh, Yu‐Han Chiu

**Affiliations:** ^1^ Department of Statistics and Actuarial Science University of Waterloo Waterloo Ontario Canada; ^2^ Department of Epidemiology and Biostatistics Harvard T.H. Chan School of Public Health Boston Massachusetts USA; ^3^ Department of Epidemiology Brown University School of Public Health Providence Rhode Island USA; ^4^ Center for Gerontology and Healthcare Research Brown University School of Public Health Providence Rhode Island USA

**Keywords:** conditional average treatment effect, conditional influence functions, data integration, generalizability, nested trial design, randomized controlled trials

## Abstract

Randomized controlled trials often enroll participants whose characteristics differ from those of a target population. When the distribution of effect modifiers differs between trial participants and the target population, treatment effects estimated within the trial may not be directly applicable to all trial‐eligible individuals. Although substantial progress has been made in generalizing average treatment effects from randomized trials to target populations, clinical and policy decisions are often informed by conditional average treatment effects (CATEs) that characterize heterogeneity across selected effect modifiers in the target population. In this work, we provide a unified approach for estimating CATEs in a target population of trial‐eligible individuals under a nested trial design. Our approach first estimates nuisance functions using data‐adaptive methods and constructs pseudo‐outcomes from conditional influence functions. These pseudo‐outcomes are subsequently regressed on pre‐specified effect modifiers using local linear (kernel) regression to obtain a smoothed estimate of the CATE function. We establish asymptotic properties under sample splitting and cross‐fitting, and provide conditions under which first‐stage nuisance estimation has negligible impact on the final estimator. We assess the finite‐sample performance of the proposed estimator via simulation studies, and illustrate its practical use through an application to the Coronary Artery Surgery Study (CASS).

## 1 Introduction

In randomized controlled trials, the average treatment effect (ATE) is often the primary estimand. In many applications, however, investigators are interested not only in the overall treatment effect in the study population but also in how treatment effects vary across individuals with different baseline characteristics. Such treatment effect heterogeneity is commonly summarized through the conditional average treatment effect (CATE), which characterizes treatment effects as a function of selected effect modifiers [1, 2, 3, 4, 5].

Trial participants, however, are often not representative of the broader target population of trial‐eligible individuals. When key effect modifiers differ between the trial and the target population, the ATE estimated within the trial may not be directly applicable to the population in which treatment decisions are ultimately made [6, 7, 8]. For instance, the Coronary Artery Surgery Study (CASS), a randomized trial nested within a broader cohort of trial‐eligible individuals, identified prior myocardial infarction and left ventricular function as key effect modifiers of the effect of coronary artery bypass grafting versus medical therapy on mortality risk [9]. In such settings, investigators may also wish to generalize treatment effect heterogeneity estimated among trial participants to the broader population of trial‐eligible individuals.

The literature on generalizability and transportability has primarily focused on extending ATEs from randomized trials to the target population of interest [6, 7, 8, 10, 11, 12, 13, 14, 15, 16], with comparatively less attention devoted to CATEs [17]. A key consideration in this setting is distinguishing variables used to adjust for selective trial participation from variables used to define treatment effect heterogeneity. The former support the generalization of inferences from the trial to the target population, while the latter define the CATE of scientific or clinical interest. This distinction parallels the separation between confounding adjustment and effect modification in observational studies. In practice, investigators may wish to adjust for a rich set of variables for trial participation while summarizing treatment effect heterogeneity using a smaller set of clinically meaningful effect modifiers, particularly when certain variables may be considered inappropriate or inequitable for guiding treatment recommendations (e.g., socioeconomic or insurance status).

However, even when the selected subset of effect modifiers is well‐justified, a CATE estimated from a nested trial may not be directly applicable to a broader population if the remaining effect modifiers have different conditional distributions between the trial and the broader population. Intuitively, a CATE defined by conditioning on only a subset of effect modifiers still averages over the remaining effect modifiers. If the distribution of these remaining effect modifiers, conditional on the variables defining the CATE, differs between populations, then the CATE estimated among trial participants may differ from the corresponding CATE in the target population.

Existing approaches for generalizing CATEs to a target population have focused on subgroup‐specific ATEs defined by a small number of discrete (or discretized) covariates, or on regression‐based methods that rely on parametric models for the nuisance functions and the CATE function [18, 19]. These approaches may be difficult to apply when the CATE is defined as a function of continuous covariates or multiple discrete covariates. In parallel, a growing literature has developed semiparametric and machine learning methods for CATE estimation [2, 20, 21, 22, 23, 24, 25, 26, 27, 28]. However, these methods typically assume that the study population coincides with the target population and therefore do not directly address selective trial participation or generalizability of findings to a broader population.

Unlike existing work that considers generalizability and flexible estimation of CATEs in separate settings, we build on conditional influence function‐based methods [21] to provide a unified framework for generalizing CATEs from a randomized trial to a target population of trial‐eligible individuals under a nested trial design [13, 29]. The proposed approach integrates flexible CATE estimation with semiparametric techniques for generalizability analyses. In the first stage, we estimate the necessary nuisance components using flexible data‐adaptive (machine learning) methods and use these estimates to construct pseudo‐outcomes whose conditional expectation corresponds to the target population CATE. In the second stage, we regress the pseudo‐outcomes on the selected effect modifiers using local linear (kernel) regression to obtain a smoothed estimate of the CATE function. This approach allows continuous and multivariate effect modifiers without requiring discretization or a parametric model for the CATE function. We establish asymptotic properties of our estimator under sample splitting and cross‐fitting, evaluate its finite‐sample performance via simulations, and present an application estimating the CATE of coronary artery bypass grafting versus medical therapy on mortality risk in CASS, conditional on prior myocardial infarction and left ventricular function.

This paper is organized as follows. In Section 2, we describe the observed data structure and define the causal estimand of interest, the CATE, in a nested trial setting. In Section 3, we state assumptions sufficient to identify and generalize the causal estimand from the trial to all trial‐eligible individuals. In Sections 4 and 5, we describe a debiased machine learning approach for generalizing the CATE to all trial‐eligible individuals, based on conditional influence functions, and study its asymptotic properties and associated inference procedures. In Sections 6, we investigate the empirical performance of the proposed estimator through extensive simulation studies, and in Section 7 we apply it in an analysis of data from CASS. Section 8 provides concluding remarks and discussion.

## 2 Notation and Setup

Consider a trial nested in a cohort of trial‐eligible individuals, such as when trial participants are recruited from a broader registry of trial‐eligible individuals [29]. Let $A=\left\{a,a^{\prime }\right\}$ denote the set of treatment values evaluated in the randomized trial, and S denote an indicator of trial participation (i.e., S=1 for randomized individuals; S=0 for nonrandomized trial‐eligible individuals).

Similar to other generalizability analyses, we assume that baseline covariate information (X) is available from all individuals in the cohort (S∈{0,1}), whereas treatment assignment (A) and outcome (Y) information is only available from randomized trial participants (S=1). Treatment and outcome data are not required for nonrandomized individuals (S=0), as they may be unavailable or deemed less reliable. To simplify exposition, we assume full adherence to assigned treatment, no measurement error, and no dropout in the trial.

We model the data as independent and identically distributed realizations of the random tuple $O_{i}=\left(X_{i},S_{i},S_{i}\times A_{i},S_{i}\times Y_{i}\right)∼F$, $i=1,\ldots ,n_{s=1},n_{s=1}+1,\ldots ,n_{s=1}+n_{s=0}$, where $n_{s=1}$ denotes the number of randomized individuals, $n_{s=0}$ the number of nonrandomized individuals, and $n=n_{s=1}+n_{s=0}$ the total number of trial‐eligible individuals in the cohort. For a random variable Z, let ${\mathbb{P}}_{n}\left(Z\right)=n^{-1}\sum_{i=1}^{n}Z_{i}$, and let $f\left(z_{2}\right)$ and $f\left(z_{2}|z_{1}\right)$ denote the marginal and conditional distributions (density or mass function) of $Z_{2}$, evaluated at $z_{2}$, with the latter conditional on $Z_{1}=z_{1}$. Henceforth, subscripts i are omitted unless needed. The causal directed acyclic graphs in Figure A.1 in the Appendix A in Supporting Information illustrate a possible data generating mechanism for our running example.

Our target of inference is the CATE in the population of trial‐eligible individuals, which includes both randomized and nonrandomized trial‐eligible individuals. Let V denote a set of baseline covariates within X identified a priori as key effect modifiers of interest. For simplicity we focus on V∈ℝ, but the results herein can be generalized to $V\in {\mathbb{R}}^{d}$, for d>1. Let $Y^{a}$ denote the potential outcome under treatment a∈A. For any pair of treatments $a,a^{\prime }\in A$, the CATE is defined as:

$$E\left(Y^{a}-Y^{a^{\prime }}|V\right),$$

which characterizes how treatment effects vary across values of V in the target population.

## 3 Generalizing Conditional Average Treatment Effects

Consider the conditional mean potential outcome $E\left(Y^{a}|V\right),a\in A$, from which the CATE is defined. We posit the following conditions [13], which are sufficient for nonparametric identification of the conditional mean potential outcome and the CATE in the target population, and are assumed to hold for each a∈A: (a) *Consistency*: For individuals who receive treatment A=a, their observed outcome equals the potential outcome under a (i.e., if A=a, then $Y=Y^{a}$). This formulation implicitly assumes that trial participation does not affect the outcome except through treatment (e.g., no trial engagement effect; [29]); (b) *Conditional exchangeability over treatment* A *in the trial*: Among randomized individuals (S=1), treatment assignment is independent of potential outcomes conditional on baseline covariates X, that is, $Y^{a}╨A|\left(X,S=1\right)$; (c) *Positivity of treatment assignment in the trial*: For all covariate values x in the support of X among randomized individuals S=1, the probability of assignment to each treatment level is positive, that is, Pr(A=a|X=x,S=1)>0, ∀x∈support(X|S=1); (d) *Conditional exchangeability (generalizability) over trial participation* S: Conditional on baseline covariates X, the distribution of the potential outcomes is the same across subsets of the target population defined by trial participation, that is, $Y^{a}╨S|X$; and (e) *Positivity of trial participation*: For all covariate values x in the support of X in the target population, the probability of trial participation is positive, that is, Pr(S=1|X=x)>0, ∀x∈support(X).

For well‐defined treatment interventions in randomized trials, conditions (a)–(c) are generally expected to be satisfied by design. Conditions (d) and (e) allow one to extend inferences about both ATE and CATE from individuals in the nested trial to the target population of all trial‐eligible individuals. Of note, conditions (b) and (d) can be relaxed to the weaker conditional mean exchangeability conditions $E\left(Y^{a}|X,A,S=1\right)=E\left(Y^{a}|X,S=1\right)$ and $E\left(Y^{a}|X,S=1\right)=E\left(Y^{a}|X\right)$, respectively [11, 30]. These conditions are sufficient to identify the potential outcome means but, unlike conditions (b) and (d), their plausibility cannot be assessed graphically using, for example, single‐world intervention graphs [31].

The probability of treatment in the trial, Pr(A=1|X=x,S=1), is usually known by design and can be treated as known or estimated using a parametric model (the latter has been shown to improve the precision of inverse probability weighted estimators, particularly when the model includes covariates predictive of the outcome; [13, 32]). In contrast, the trial selection mechanism and outcome models, Pr(S=1|X) and E(Y|A=a,X,S=1) are generally unknown and may be complex, motivating the use of data‐adaptive methods for their estimation. Unlike observational studies, where adjustment is needed for confounding between treatment and outcome, here X serves to account for selective participation into the trial. Under the identifiability conditions listed above, the conditional mean potential outcome under a in the target population of all trial‐eligible individuals given V, $E\left(Y^{a}|V\right)$, is identified by the following observed data functional:

ψ(a;V)≔E[E(Y|A=a,X=x,S=1)∣V].

As such, the CATE, $E\left(Y^{a=1}|V\right)-E\left(Y^{a=0}|V\right)$, is identified as:

$$\begin{array}{ll} & \psi \left(a=1;V\right)-\psi \left(a=0;V\right)=E\left[E\left(Y|A=1,X,S=1\right)|V\right]\\ & \;-E\left[E\left(Y|A=0,X,S=1\right)|V\right]. \end{array}$$

For notational simplicity, we define θ(V)≔ψ(a=1;V)−ψ(a=0;V). In the remainder of the paper, we focus on the estimation of the CATE, identified by the observed data functional θ(V), for the target population of all trial‐eligible individuals.

## 4 Conditional Influence Function for the Conditional Average Treatment Effects

Consider the observed data functional μ(a)≔E[E(Y|A=a,X,S=1)], which identifies the marginal mean potential outcome $E\left(Y^{a}\right).$ The influence function for μ(a) [13], can be viewed as the Gateaux derivative of μ(a) in the direction of a perturbation of the underlying probability distribution F [33, 34], which motivates the following conditional influence function.

Now we consider the conditional mean potential outcome under treatment level a (for a∈A), which is identified by ψ(a;V)=E[E(Y|A=a,X,S=1)∣V]. The corresponding conditional influence function yields conditionally Neyman orthogonal estimating equations for the CATE, and provides a principled basis for constructing pseudo‐outcomes that inherit desirable robustness properties [21]. Let $F_{V}$ denote the conditional cumulative distribution function (CDF) of O given V, and define the conditional influence function $\phi_{V}\left(a;O\right)$ to be the *Gateaux derivative* of $\psi \left(a;V\right)=\psi \left(a;F_{V}\right)$ with respect to $F_{V}$ at ϵ=0 as the following:

$${\left.\frac{d\psi \left(a;F_{\epsilon ,V}\right)}{d\epsilon }\right|}_{\epsilon =0}=\int \phi_{V}\left(a;O\right){\mathrm{dH}}_{V}\left(o\right),\;E\left[\phi_{V}\left(a;O\right)|V\right]=0,$$

where $H_{V}$ is a conditional CDF that may be different than $F_{V}$, and $F_{\epsilon ,V}=\left(1-\epsilon \right)F_{V}+\epsilon H_{V}$ for some ϵ∈[0,1]. The Gateaux derivative yields the following expression for the conditional influence function of ψ(a;V), with a proof provided in Appendix B in Supporting Information.

Theorem 1 *The conditional influence function of* ψ(a;V) *is given by:*

$$\begin{array}{ll} \phi_{V}\left(a;O\right) & =\left[\frac{S\cdot I\left(A=a\right)}{\mathrm{Pr}\left(S=1|X\right)\mathrm{Pr}\left(A=a|X,S=1\right)}\left\{Y-\gamma \left(Z\right)\right\}\right.\\ & \;\left.+\gamma \left(a,X,S=1\right)-\psi \left(a;V\right)\right]. \end{array}$$

*where* Z=(A,X,S), γ(Z)=E(Y|A,X,S) *and* γ(a,X,S=1)=E(Y|A=a,X,S=1).

Corollary 1 *The conditional influence function of* θ(V) *is given by:*

(1) $$\begin{array}{ll} \phi_{\text{CATE}}\left(O\right) & =\left[\underset{\alpha \left(Z\right)}{\underbrace{\frac{S\cdot \left(2A-1\right)}{\mathrm{Pr}\left(S=1|X\right)f\left(A|X,S=1\right)}}}\left\{Y-\gamma \left(Z\right)\right\}\right.\\ & \;\left.+\left\{\underset{m\left(X,S=1;\gamma \right)}{\underbrace{\gamma \left(a=1,X,1\right)-\gamma \left(a=0,X,S=1\right)}}\right\}-\theta \left(V\right)\right]\\ & =\alpha \left(Z\right)\left\{Y-\gamma \left(Z\right)\right\}+m\left(X,S=1;\gamma \right)-\theta \left(V\right) \end{array}$$

*where* $\alpha \left(Z\right)≔\frac{S\cdot \left(2A-1\right)}{\mathrm{Pr}\left(S=1|X\right)f\left(A|X,S=1\right)};\;\text{and}\;m\left(X,S=1;\gamma \right)=\gamma \left(a=1,X,S=1\right)-\gamma \left(a=0,X,S=1\right)$.

The term α(Z) in (1) is the conditional Riesz representer such that θ(V)=E[α(Z)γ(A,X,S=1)∣V], where θ(V)=E[m(X,S=1;γ)|V]. This is the conditional analogue of the usual influence function representation for a marginal parameter: just as the marginal expectation of the uncentered influence function (i.e., the influence function plus the parameter) for the ATE recovers the marginal parameter, the conditional expectation of the corresponding uncentered conditional influence function recovers the CATE. Similar to influence functions for marginal quantities, the conditional influence function (1) can be used to construct CATE estimators when data‐adaptive methods are used to estimate nuisance functions. Under regularity conditions outlined below, the estimation error in these nuisance functions is asymptotically negligible, so the proposed estimator converges at the same rate as if the nuisance functions were known. As noted in Chernozhukov et al. [21], unlike the influence functions for marginal quantities, conditional influence functions cannot be used to facilitate efficiency comparisons between different localization procedures (e.g., kernel smoothing methods, nearest‐neighbor or series‐based methods) when V is continuous. This limitation arises because the form of localization applied can affect the asymptotic variance. Here, we focus on local linear (kernel) regression for estimating the CATE.

## 5 Debiased Machine Learning for the Conditional Average Treatment Effects

We adapt a debiased machine learning approach based on local linear regression. In line with prior work (e.g., [21, 35]), our results require mean‐square convergence rates for the first‐stage nuisance estimators estimated using data‐adaptive methods. To guard against overfitting of the nuisance functions α(Z) and m(Z) as defined above, the algorithm utilizes sample splitting and cross‐fitting.

To estimate θ(V), we perform localized regression around V. Following Chernozhukov et al. [21] (see also [28, 35, 36]), we apply sample splitting and cross‐fitting to estimate the nuisance functions before the second‐stage nonparametric local linear regression. Let K(u) be a kernel with ∫K(u)du=1 and let h>0 denote a bandwidth. The algorithm is given as follows:

1. First, partition the data into L disjoint subsets of equal size ($I_{l},$ l=1,…,L). For each fold l=1,…,L, and for all $i\in I_{l}$, we estimate
$$\hat{\xi }\left(O_{i};{\hat{\alpha }}_{-l},{\hat{\gamma }}_{-l}\right)={\hat{\alpha }}_{-l}\left(Z_{i}\right)\left\{Y_{i}-{\hat{\gamma }}_{-l}\left(Z_{i}\right)\right\}+{\hat{m}}_{-l}\left(X_{i},S_{i}=1,{\hat{\gamma }}_{-l}\right),$$
  where the nuisance functions are estimated using $I_{-l}$, that is, observations that are not in $I_{l}$. Note that f(A|X,S=1) and γ(A,X,S=1) are estimated using only individuals with S=1 in $I_{-l}$, while Pr(S=1|X) is estimated using all individuals in $I_{-l}.$
2. $\hat{\xi }\left(O_{i}\right)≔\hat{\xi }\left(O_{i};{\hat{\alpha }}_{-l},{\hat{\gamma }}_{-l}\right)$ are then regressed on $V_{i}$ via locally linear smoother [37, 38, 39, 40]. The locally linear estimator is given by:
(2) $$\hat{\theta }\left(v\right)=\underset{\theta ,\delta }{\arg\;\min}\;\sum_{l=1}^{L}\sum_{i\in I_{l}}\left[{\left\{\hat{\xi }\left(O_{i}\right)-\theta -{\left(V_{i}-v\right)}^{T}\delta \right\}}^{2}K_{h}\left(V_{i}-v\right)\right],$$
  where $K_{h}\left(u\right)=h^{-1}K\left(u/h\right)$ (more generally, $K_{h}\left(u\right)=h^{-d}K\left(u/h\right)$ for d>1).

### 5.1 Asymptotic Properties

Following Chernozhukov et al. [21] (see also [41]), we adopt the following conditions on the kernel K(u) and the relevant functionals:

Assumption 1 (Regularity conditions).

- K *is a continuous symmetric probability density with support* [−1,1] *such that* ∫K(u)du=1 *and* K(u) *and* $K\left(u\right)u^{2}$ *are bounded;*
- *The bandwidth* $h=h_{n}$ *satisfies* nh→∞, h→0 *;*
- $∥\hat{\gamma }-\gamma ∥=o_{p}\left(h^{1/2}\right)$, $∥\hat{\alpha }-\alpha ∥=o_{p}\left(h^{1/2}\right)$, $\sqrt{n}∥\hat{\gamma }-\gamma ∥∥\hat{\alpha }-\alpha ∥=o_{p}\left(h^{1/2}\right).$

Conditions I and II of Assumption 1 are standard in nonparametric regression. In particular, Condition II requires that the “local sample size” must increase with sample size while bandwidth h must go to zero [41, 42]. Condition III plays a central role in the analysis and requires that the mean square product of the nuisance estimators converges to zero at a rate faster than $h^{1/2}n^{-1/2}$, which is slightly stronger than the usual $n^{-1/2}$ product rate condition by an additional factor of $h^{1/2}$.

Theorem 2 *Under the aforementioned conditions* (Assumption 1),

$$\sqrt{\mathrm{nh}}\left[\hat{\theta }\left(v\right)-\tilde{\theta }\left(v\right)\right]\overset{p}{\to }0,$$

*where* $\tilde{\theta }\left(v\right)$ *is an oracle estimator that would be available if the nuisance functions were known. The proof of* Theorem 2 *is presented in* Appendix C in Supporting Information. *For* $V\in {\mathbb{R}}^{d}\;\left(d>1\right)$, *the results follow analogously if (1)* Assumption 1‐I *changes to “K is a continuous, symmetric multivariate probability density on* ${\mathbb{R}}^{d}$ *with* ∫K(u)du=1, *compact support (*e.g., ${\left[-1,1\right]}^{d}$ *), and such that* K(u) *and* ${\left\|u\right\|}^{2}K\left(u\right)$ *are bounded”; (2)* nh *in* Assumption 1‐II *changes to* ${\mathrm{nh}}^{d}$ [41]*; and (3)* $h^{1/2}$ *in* Assumption 1‐III *changes to* $h^{d/2}$ [21]*; then it follows that* $\sqrt{{\mathrm{nh}}^{d}}\left[\hat{\theta }\left(V\right)-\tilde{\theta }\left(V\right)\right]\overset{p}{\to }0$.

Theorem 2 implies that the estimation errors in the nuisance functions are asymptotically negligible, so the proposed estimator converges at the same rate as if the nuisance functions were known. Moreover, following Fan [43] and Fan et al. [40], $E\left[{\left\{\tilde{\theta }\left(v\right)-\theta \left(v\right)\right\}}^{2}\right]=O\left(1/\mathrm{nh}+h^{4}\right)$, and $\sqrt{\mathrm{nh}}\left(\tilde{\theta }\left(v\right)-\theta \left(v\right)-B_{h}\left(v\right)\right)$ converges in distribution to a mean‐zero normal random variable with finite variance, where $B_{h}\left(v\right)={\theta^{\prime }}^{\prime }\left(v\right)\left(h^{2}/2\right)\int u^{2}K\left(u\right)\mathrm{du}$; for d>1, replacing nh by ${\mathrm{nh}}^{d}$ yields the corresponding mean squared error and asymptotic normality results [41, 44].

Theorem 3 *Under* Assumption 1, *and the additional smoothness conditions below:*

- *The functional* θ(v) *is twice continuously differentiable with bounded derivatives;*
- *The density* f(v) *is continuously differentiable and bounded away from zero in a neighborhood of* v. *Moreover, the conditional density of* ξ(O;α,γ) *given* V=v *exists and is continuous in* (ξ,v), *where* ξ(O;α,γ)=α(Z){Y−γ(Z)}+m(X,S=1;γ);
*and additionally if* ${\mathrm{nh}}^{5}=O\left(1\right)$, *then:*
$$\sqrt{\mathrm{nh}}\left\{\hat{\theta }\left(v\right)-\theta \left(v\right)-B_{h}\left(v\right)\right\}\overset{d}{\to }N\left\{0,\frac{\sigma^{2}\left(v\right)\int K{\left(u\right)}^{2}\mathrm{du}}{f\left(v\right)}\right\},$$
*where* $B_{h}\left(v\right)={\theta^{\prime }}^{\prime }\left(v\right)\left(h^{2}/2\right)\int u^{2}K\left(u\right)\mathrm{du},$ *and* $\sigma^{2}\left(v\right)=\mathrm{Var}\left\{\xi \left(O;\alpha ,\gamma \right)|V=v\right\}$.

For $V\in {\mathbb{R}}^{d}\;\left(d>1\right)$, the asymptotic normality results can be generalized following Theorem 2.7 of Li and Racine [41].

As in standard nonparametric regression, the estimator is consistent but not exactly centered at θ(v); instead, it exhibits a bias denoted $B_{h}\left(v\right)$ [41, 45]. When ${\mathrm{nh}}^{5}=O\left(1\right)$, under our stated conditions, the remainder term is asymptotically negligible under the normalization $\sqrt{\mathrm{nh}}$ and therefore the limiting distribution can equivalently be expressed in terms of $\theta^{*}\left(v\right)=\theta \left(v\right)+B_{h}\left(v\right)$. Consequently, the estimator is centered at a smoothed version of the effect curve, $\theta^{*}\left(v\right)$, which, as noted in Wasserman [45], complicates the construction of confidence intervals. Following Wasserman [45], Kennedy et al. [46] and related work, we quantify uncertainty in $\hat{\theta }\left(v\right)$ using confidence intervals centered at the smoothed, data‐dependent parameter $\theta^{*}\left(v\right)$.

Define $\beta ={\left(\theta ,\delta \right)}^{T},$ where θ denotes the local intercept and δ the local slope parameter in the local linear estimator defined in (2). In Appendix C in Supporting Information, we show that the influence function for β can be found to be equal to

$$\varphi \left(O\right)=D^{-1}\left[g\left(V\right)K_{h}\left(V-v\right)\left\{\xi \left(Z;\eta \right)-g{\left(V\right)}^{T}\beta \right\}\right],$$

where $g\left(V\right)={\left(1,V-v\right)}^{T}$ and $D=E\left[g\left(V\right)K_{h}\left(V-v\right)g{\left(V\right)}^{T}\right].$ Based on this result, pointwise Wald‐style 95% confidence intervals for the smoothed CATE can be constructed with $\hat{\theta }\left(v\right)\pm 1.96\hat{\sigma }/\sqrt{n},$ where ${\hat{\sigma }}^{2}$ is the (1,1)‐th element of sandwich variance estimate ${\mathbb{P}}_{n}\left[\hat{\varphi }\left(O\right)\hat{\varphi }{\left(O\right)}^{T}\right],$ where $\hat{\varphi }\left(O\right)$ defined analogously to φ(O), with the nuisance functions replaced by their estimators and D replaced by ${\mathbb{P}}_{n}\left[g\left(V\right)K_{h}\left(V-v\right)g{\left(V\right)}^{T}\right].$ Corresponding uniform confidence bands can also be constructed following the methods of Lee et al. [26].

As such, we should interpret the proposed local linear estimator as a consistent estimator of $\theta^{*}\left(v\right)$, which equals a kernel‐weighted average of the function θ(⋅) in a small neighborhood around v, and can be viewed as a kernel‐smoothed version of the true CATE function [45]. In this sense, for a fixed bandwidth, the estimator targets a local approximation rather than the exact pointwise value at v. As the bandwidth decreases with increasing sample size, the neighborhood around v shrinks and $\theta^{*}\left(v\right)$ converges to θ(v).

## 6 Simulation Studies

We simulated cohorts of n=(2500,5000) individuals and varied the number of randomized participants $n_{s=1}=\left(125,500,1000\right)$, to assess performance across different proportions of trial participants relative to the target population of trial‐eligible individuals. Following Dahabreh et al. [13], we generated covariates $\left(X_{1},X_{2},X_{3}\right)$, trial participation (S), random treatment assignment among trial participants (A) using simple Bernoulli randomization, and outcome of interest (Y), considered under both binary and continuous settings. Our target was the CATE given by $E\left(Y^{a=1}-Y^{a=0}|X_{2}\right)$. Details of the simulation setup and additional results for the continuous case are provided in Appendix D in Supporting Information.

We compared the proposed estimator with a naive alternative that used the same first‐stage nuisance function estimates and pseudo‐outcome construction, but replaced local linear regression with a simple linear regression of the pseudo‐outcome on $X_{2}$. Performance was evaluated against the true CATE function. For binary outcomes, the true underlying CATE function does not admit a closed‐form expression and was therefore approximated via Monte Carlo integration of size $n=10^{7}$; for continuous outcomes, the true underlying CATE function is available in closed form. In the continuous‐outcome setting, we also compared the proposed estimator with an oracle estimator that used the same first‐stage nuisance function estimates but specified the second‐stage model using the true functional form.

Nuisance functions were estimated using the Super Learner ensemble, which uses cross‐validation to select the best convex combination of predictions from a library of candidate algorithms [47]. The library included generalized linear models and its variants (SL.glm, SL.glm.interaction), gradient boosting (SL.xgboost), generalized additive models with smoothing splines (SL.gam), multivariate adaptive regression Splines (SL.earth), neural networks (SL.nnet) and random forest (SL.ranger). Note that the convergence rate conditions in Assumption 1 applies to the nuisance function estimators produced by Super Learner, rather than to every candidate learner individually. The Super Learner's oracle property ensures that the ensemble performs at least as well as the best‐performing learner(s) in the library [47]. In practice, we recommend including both learners informed by substantive knowledge that can achieve fast convergence rates when the true nuisance functions are well approximated (e.g., SL.glm, SL.glm.interaction), and more flexible learners that can accommodate greater model complexity and may achieve sufficiently fast rates under structural assumptions on the nuisance functions, such as smoothness or sparsity (e.g., SL.gam, SL.ranger; [36, 48]), as noted in previous literature [46, 49, 50].

For our proposed estimator, the CATEs were estimated using local linear regression, with the bandwidth parameter selected via cross‐validation [46, 51]. Alternatively, one may use a direct plug‐in bandwidth selector such as that of Ruppert et al. [52], but such selectors have been observed to oversmooth in practice [53]. Performance of the estimator was summarized over the 1000 simulated datasets using integrated absolute bias and root mean squared error (RMSE). We also assessed finite‐sample inference by computing integrated coverage probabilities based on the proposed sandwich variance estimator.

### 6.1 Simulation Results

Simulation results are presented in Table 1 and Figure 1, with additional results for the continuous outcome provided in Appendix D in Supporting Information. For both binary and continuous outcomes, the proposed local linear regression estimator substantially reduced bias relative to the naive regression approach. Across all sample sizes for the continuous outcome, the oracle linear regression estimator, which correctly specifies the true CATE function, exhibited the smallest integrated absolute bias and achieved coverage probabilities closest to the nominal level. With the exception of the naive regression estimator, the integrated absolute bias of the other estimator(s) decreased as $n_{s=1}$ increased for a fixed cohort size n.

**TABLE 1:** Integrated bias, integrated RMSE, and integrated 95% coverage probability (CP) for binary outcome.

Method	Integrated ∣Bias∣	Integrated RMSE	Integrated CP
$n_{s=1}=125,\;n=2500$			
Local linear regression	0.022	0.345	92
Linear regression (naive)	0.096	0.322	91
$n_{s=1}=500,\;n=2500$			
Local linear regression	0.020	0.167	92
Linear regression (naive)	0.101	0.168	77
$n_{s=1}=1000,\;n=2500$			
Local linear regression	0.019	0.085	92
Linear regression (naive)	0.102	0.122	47
$n_{s=1}=125,\;n=5000$			
Local linear regression	0.019	0.351	92
Linear regression (naive)	0.097	0.327	91
$n_{s=1}=500,\;n=5000$			
Local linear regression	0.017	0.217	91
Linear regression (naive)	0.102	0.187	78
$n_{s=1}=1000,\;n=5000$			
Local linear regression	0.016	0.120	93
Linear regression (naive)	0.102	0.140	62

*Note:* For the $n_{s=1}=125$ scenarios, the most extreme 5% of values were trimmed.

**FIGURE 1:**
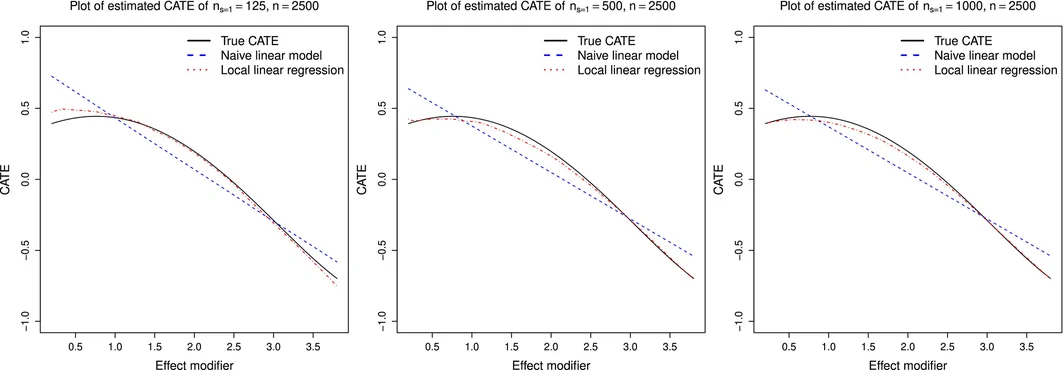
Comparison of proposed estimator with naive estimator for n=2500 across trial participation size $n_{s=1}=\left(125,500,1000\right)$ for binary outcome. 95% coverage probability across values of effect modifier ($X_{2}$) is shown for the proposed estimator.

In additional simulation studies reported in Appendix D in Supporting Information, we compared our proposed estimator with an estimator based only on available trial data. For binary outcomes, the trial‐only approach consistently produced biased estimates of the CATE, as all covariates acted as effect modifiers on the risk‐difference scale in the data‐generating mechanism across all scenarios considered. For continuous outcomes, substantial bias arose when multiple effect modifiers were present but only a subset was used to estimate the CATE (see Appendix D in Supporting Information for details).

The confidence intervals for the proposed estimator performed reasonably well, although their coverage probabilities were slightly below the nominal level for continuous outcomes (see Appendix D in Supporting Information) and closer to nominal for binary outcomes. For instance, when $n_{s=1}=1000$ and n=2500, the integrated coverage probability was 92% for the binary outcome and 88% for the continuous outcome. This modest under‐coverage may reflect finite‐sample smoothing bias in the estimator. Since inference targets a smoothed version of the CATE rather than the exact underlying curve, the reported intervals are centered on the smoothed parameter, and the remaining bias may still affect coverage in finite samples. As an approximate check on centering, we also evaluate coverage against the average estimated CATE across simulations in place of the true CATE. This yields coverage between 93%–95% for $n_{s=1}=\left(500,1000\right)$ and 90%–91% for $n_{s=1}=125$, with similar patterns across both outcome types, suggesting that the intervals generally have appropriate width but are shifted by finite‐sample bias.

## 7 Coronary Artery Surgery Study Data Analysis

### 7.1 CASS Design and Data

The Coronary Artery Surgery Study (CASS; [9, 54]) was a randomized trial nested within a cohort of trial‐eligible patients with stable ischemic heart disease. The trial compared coronary artery bypass grafting plus medical therapy (hereafter “surgery”) against medical therapy alone. Recruitment took place between August 1975 and May 1979, with follow‐up for mortality recorded through December 1996.

Our analysis included trial‐eligible patients, a subset of whom were randomized; among the randomized group, no censoring was observed during the first 10 years of follow‐up. Baseline covariate information is available for all individuals in the trial‐eligible cohort (S∈0,1), whereas treatment assignment and outcome information are restricted to the randomized trial participants (S=1), as treatment status in the nonrandomized cohort is not defined in the same way as the randomized treatment assignment [13]. The analytic sample consists of 1686 patients, including 731 randomized patients (368 to surgery and 363 to medical therapy) and 955 nonrandomized patients, and is identical to that used in Robertson et al. [55].

### 7.2 Baseline Characteristics

Baseline characteristics are summarized in Table 2. In general, nonrandomized patients were more likely to use beta‐blockers and, on average, had higher left main coronary percent obstruction and left ventricular wall scores. If these characteristics are treatment effect modifiers, beyond those previously identified, then treatment effects estimated using only trial participants may not be directly applicable to the broader population of trial‐eligible individuals. This possibility motivates the proposed approach for extending inferences beyond the randomized sample.

**TABLE 2:** Baseline characteristics in CASS.

	S=1	S=0
Age	51.17 (7.32)	50.89 (7.73)
History of angina	567 (77.6)	760 (79.6)
Taken beta‐blocker regularly	315 (43.1)	508 (53.2)
Taken diuretic regularly	113 (15.5)	145 (15.2)
Ejection fraction	60.35 (12.91)	60.16 (12.25)
Employed full‐time	497 (68.0)	632 (66.2)
Type of job		
High physical labor job	293 (40.1)	340 (35.6)
Low mental labor job	264 (36.1)	320 (33.5)
High mental labor job	174 (23.8)	295 (30.9)
Left ventricular wall score	7.37 (2.84)	7.07 (2.69)
Taken nitrates regularly	401 (54.9)	528 (55.3)
History of MI	437 (59.8)	549 (57.5)
Female	72 (9.8)	87 (9.1)
Smoking status		
Never smoked	116 (15.9)	157 (16.4)
Former smoker	321 (43.9)	451 (47.2)
Current smoker	294 (40.2)	347 (36.3)
High limitation of activities	338 (46.2)	441 (46.2)
High recreational activity	447 (61.1)	616 (64.5)
Confirmed hypertension	226 (30.9)	260 (27.2)
Diabetes status		
No diabetes	653 (89.3)	873 (91.4)
Uncertain diabetes	20 (2.7)	23 (2.4)
Confirmed diabetes	58 (7.9)	59 (6.2)
LMCA percent obstruction	3.53 (10.80)	5.76 (14.50)
PLMA percent obstruction	35.67 (37.49)	39.14 (38.73)
Any diseased proximal vessels	452 (61.8)	608 (63.7)
Systolic blood pressure	130.31 (18.06)	129.80 (18.23)

*Note:* Continuous variables are presented as mean (standard deviation), and binary variables as count (percentage). S=1 denotes the randomized group; S=0 denotes the nonrandomized group.

Abbreviations: LMCA, left main coronary artery; MI, myocardial infarction; PLMA, proximal left anterior artery.

Figure 2 presents kernel density estimates of the predicted probabilities of trial participation for randomized and nonrandomized individuals. The distributions show substantial overlap in estimated trial participation probabilities, with the smallest estimated probability among randomized individuals being approximately 0.15 (well above zero). These findings are consistent with adequate covariate support between randomized and nonrandomized individuals, supporting the proposed extension of trial inferences to the target population of all trial‐eligible individuals.

**FIGURE 2:**
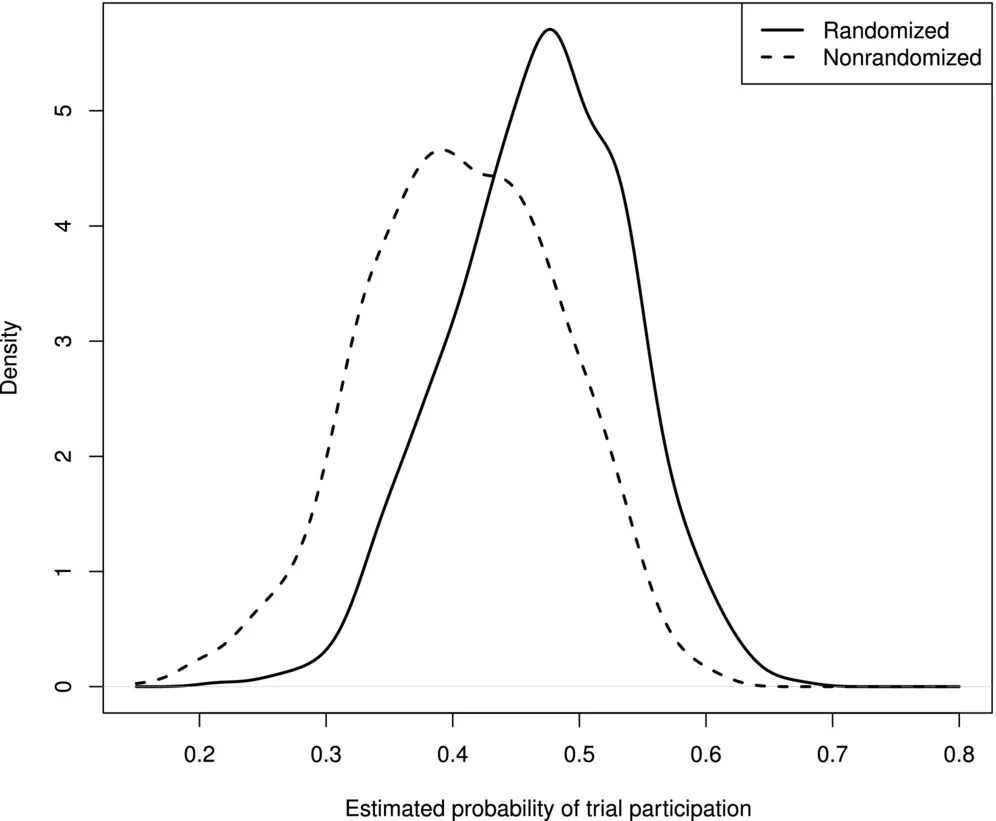
Kernel densities of the estimated probabilities of trial participation from CASS.

### 7.3 Estimands

Prior work has highlighted both prior myocardial infarction and measures of left ventricular function as important sources of heterogeneity in the effect of coronary artery bypass grafting versus medical therapy on mortality risk [9]. Motivated by this evidence, we estimated CATEs, expressed as 10‐year mortality risk differences comparing surgery versus medical therapy in the population of all trial‐eligible individuals, as functions of baseline ejection fraction and stratified by history of myocardial infarction.

### 7.4 Model Specification

The nuisance function models adjusted for all covariates considered to account for selective trial participation, including age, sex, employment status, smoking status, baseline recreational activity level, severity of angina, history of previous myocardial infarction, percent obstruction of the proximal left anterior descending artery, number of diseased vessels, left ventricular wall motion score, systolic blood pressure, diabetes status, ejection fraction, and indicators for regular use of diuretics, nitroglycerin and beta blockers (see table 2 for details; [55, 56]).

In the data analysis, nuisance functions were estimated using the Super Learner ensemble with the same library of candidate learners as in simulation studies. The CATE functions, stratified by history of myocardial infarction and evaluated over ejection fraction values ranging from 40% to 80%, were estimated using local linear regression with the optimal bandwidth selected via cross‐validation. Pointwise uncertainty intervals were constructed using a Wald approach with the proposed sandwich variance estimator.

### 7.5 Results

Figure 3 presents the estimated target population CATEs, stratified by history of myocardial infarction and over the observed range of ejection fraction values using our proposed estimator. Overall, we found the 10‐year mortality risk difference comparing surgery versus medical therapy varied by ejection fraction within levels of myocardial infarction history. Among patients with a history of myocardial infarction, the smoothed CATE increases approximately linearly from a risk difference of about −0.27 at the lower end of the ejection fraction range to approximately 0.20 at the higher end. In contrast, among patients without prior myocardial infarction, the CATE initially decreases from around −0.26 at the lower end of the range, remains near zero over much of the mid‐range, and then increases to approximately 0.15 at the higher end.

**FIGURE 3:**
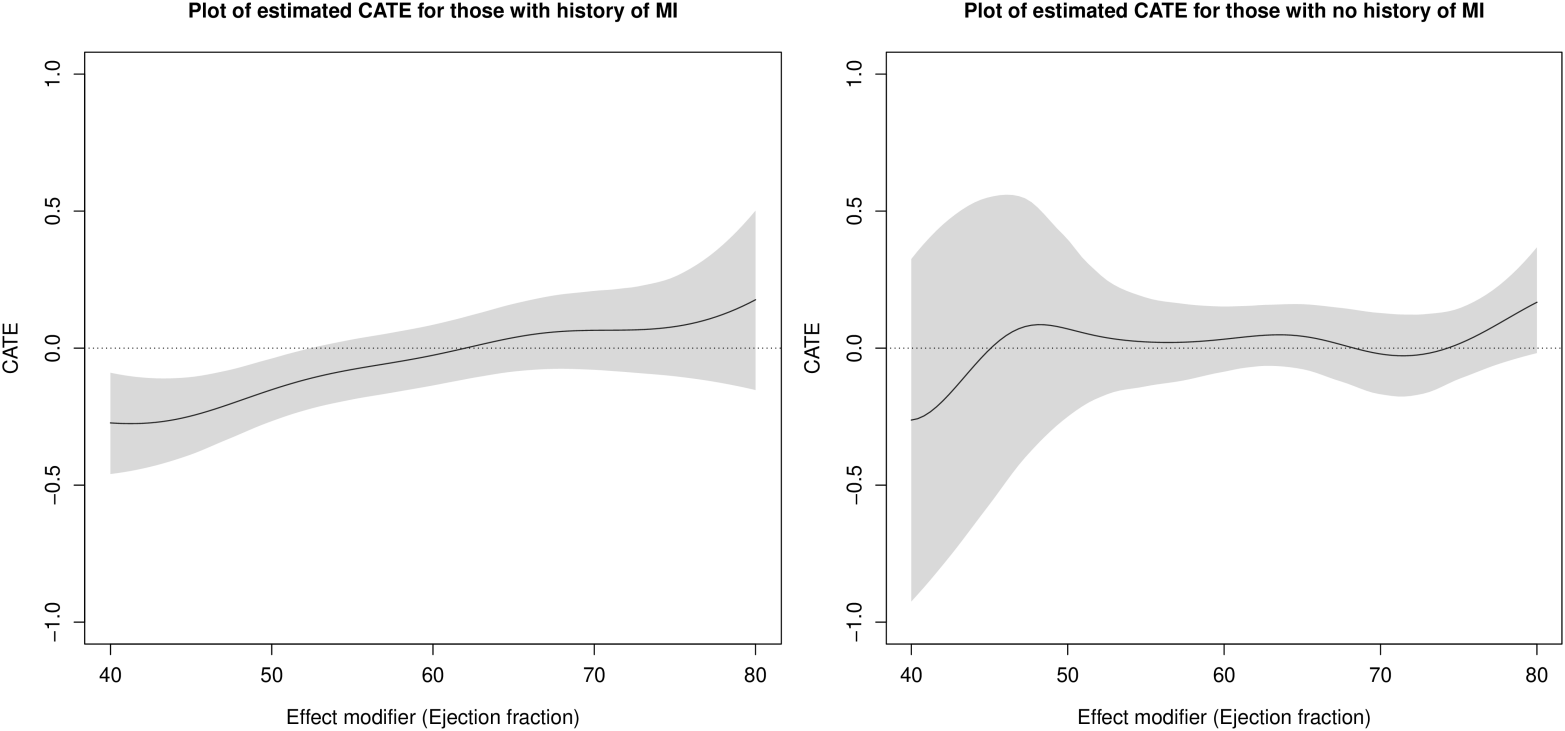
Estimated target population CATE stratified by history of myocardial infarction (MI) over the set of observed ejection fraction values from our proposed estimator.

The estimated curves were broadly consistent with prior findings in Robertson et al. [55], which analyzed the same study sample using parametric nuisance‐function models with spline‐based regression for CATE estimation. Our work extends that work by providing a more flexible estimation framework and formal asymptotic theory for generalizing CATEs from nested randomized trials to target populations. In particular, both approaches indicate a monotone increase in the CATE among patients with a history of myocardial infarction. However, among patients without a history of myocardial infarction, the proposed local linear regression approach exhibits greater uncertainty at low ejection fraction values, where observations are sparse, as reflected in wider confidence intervals relative to the spline‐based estimator of Robertson et al. [55].

To aid interpretation, we report estimates at representative values of ejection fraction spanning clinically relevant levels of cardiac function, from heart failure (40% ejection fraction) to mildly reduced function (45%) and normal function (55%; [57]). Using our proposed approach, we estimated a 10‐year mortality risk difference of −0.273 for individuals with prior myocardial infarction and an ejection fraction of 40% (95% CI: [−0.457,−0.089]), compared with −0.250 at 45% (95% CI: [−0.392,−0.107]) and −0.083 at 55% (95% CI: [−0.192,0.026]). Among individuals without a history of myocardial infarction, the corresponding estimates were −0.262 at 40% ejection fraction (95% CI: [−0.871,0.346]), −0.013 at 45% (95% CI: [−0.862,0.835]) and 0.024 at 55% (95% CI: [−0.232,0.281]).

In Appendix E in Supporting Information, we present a sensitivity analysis comparing the proposed data‐integration approach with trial‐only estimation of the CATE functions. The results from the trial‐only analysis show similar overall trends in the CATE across ejection fraction and MI groups, but the trial‐only estimates show more pronounced fluctuations in the CATE among those with no history of MI compared with the proposed data integration approach.

## 8 Discussion

In randomized trials and comparative studies, investigators often seek to understand how treatment effects vary across individuals with different baseline characteristics, typically by pre‐specifying effect modifiers based on substantive knowledge or clinical relevance. However, even when focusing on a subset of well‐justified effect modifiers, CATEs estimated from trial data alone may not be directly applied to the broader population of trial‐eligible individuals when the distribution of the other effect modifiers differs between the trial and the target population. Accounting for such differences is therefore essential for valid inference on treatment effect heterogeneity and for informing decision‐making in the target population.

Herein, we described a semiparametric framework for estimating target‐population CATEs under nested trial sampling. The proposed approach combines data‐adaptive estimation of nuisance functions and localized nonparametric regression for continuous effect modifiers. By separating variables used to adjust for selective trial participation from variables used to characterize treatment effect heterogeneity, the framework allows investigators to adjust for a rich set of covariates while restricting treatment‐effect summaries to a smaller set of clinically meaningful effect modifiers. The resulting approach enables flexible and interpretable estimation of treatment effect heterogeneity in the target population while avoiding restrictive parametric assumptions.

Our inference targets a smoothed version of the CATE, which improves stability in finite samples. Valid inference for the pointwise parameter generally requires either undersmoothing or bias correction. Undersmoothing reduces bias at the cost of inflating variance and depends on an ad‐hoc bandwidth shrinkage, whereas bias correction avoids undersmoothing but typically requires estimating additional quantities. For instance, Calonico et al. [58] developed a bias‐correction approach that improves finite‐sample performance relative to undersmoothing but requires estimation of higher‐order derivatives of the CATE function via additional local polynomial regression. Future work will investigate how these robust bias‐correction techniques can be integrated with the methods developed here to improve coverage accuracy while maintaining stability in finite samples. Additional extensions include accommodating higher‐dimensional effect modifiers and developing context‐specific CATE estimators for non‐nested trial settings.

Our algorithm uses sample splitting without explicitly stratifying on trial participation S. In settings where the randomized trial is small relative to the sample of nonrandomized trial‐eligible individuals, stratifying the sample splits by S may improve finite‐sample performance by ensuring that each fold contains a sufficient number of trial participants for estimating the trial‐specific nuisance functions. Similar stratification strategy could also be used when estimating nuisance functions with Super Learner or selecting the bandwidth in Step 2 of our algorithm via cross‐validation. We do not consider stratified sample splitting in the present work, but evaluating its finite‐sample performance is an interesting direction for future research.

## Funding

This work was supported by the Natural Sciences and Engineering Research Council of Canada (DGECR‐2023‐00455), National Institute of General Medical Sciences (R35GM154888), National Library of Medicine (R01LM013616), National Heart, Lung, and Blood Institute (R01HL136708), and Patient‐Centered Outcomes Research Institute (ME‐2021C2‐22365).

## Conflicts of Interest

The author declares no conflicts of interest.

## Supporting information


**Appendix A:** Data structure and identification of the conditional average treatment effect.
**Figure A.1:** Directed acyclic graphs (DAGs) between randomized and non‐randomized individuals: (top) all trial eligible individuals; (bottom left) individuals in S=1; (bottom right) individuals in S=0.
**Appendix B:** Proof of the conditional influence function.
**Appendix C:** Proof of Theorem 2.
**Appendix D:** Simulation details.
**Table D.1:** Integrated bias, integrated RMSE, and integrated 95% coverage probability (CP) for continuous outcome. For the $n_{s=1}=125$ scenarios, the most extreme 5% of values were trimmed.
**Figure D.2:** Comparison of proposed estimator with naive and oracle estimators across trial participation size $n_{s=1}$ = (125, 500, 1000) for n=2500 for *continuous outcome*. 95% coverage probability across values of effect modifier ($X_{2}$) is shown for the proposed estimator.
**Figure D.3:** A comparison of true CATE of interest among all trial‐eligible individuals versus true CATE of trial only participants.
**Table D.2:** Results from the local linear regression estimator based on trial only participants for binary outcome.
**Table D.3:** Results from the local linear regression estimator based on trial only participants for continuous outcome.
**Figure D.4:** A comparison of true CATE of interest among all trial‐eligible individuals versus true CATE of trial only participants.
**Table D.4:** Supplementary simulation for binary outcomes: comparison of the proposed local linear and naive linear regression estimators.
**Table D.5:** Supplementary simulation for binary outcomes: results from local linear regression estimator based on trial only participants.
**Figure D.5:** A comparison of true CATE of interest among all trial‐eligible individuals versus true CATE of trial only participants.
**Table D.6:** Supplementary simulation for continuous outcomes: comparison of the local linear, naive and oracle linear regression estimators.
**Table D.7:** Results from the local linear regression estimator based on trial only participants for continuous outcome.
**Appendix E:** Additional data application results.
**Figure E.6:** Trial only estimated CATE stratified by history of myocardial infarction (MI) over the set of observed ejection fraction values from our proposed estimator.

## Data Availability

The data that support the findings of this study are openly available in the National Heart, Lung, and Blood Institute (NHLBI) Biologic Specimen and Data Repository Information Coordinating Center (BioLINCC) at https://biolincc.nhlbi.nih.gov/studies/cass/, reference number HLB00250309a. R code to replicate simulation and application results is available on https://github.com/lw499/generalizability_cate.
