# Supplementary material for: Generalizing Conditional Average Treatment Effects From Nested Randomized Trials to Trial‐Eligible Individuals
Source: Stat Med. 2026 Oct 9;45(23-24):e70767. doi: 10.1002/sim.70767 (PMC13652670; doi:10.1002/sim.70767)
Supplement: Supplementary file 1 [file SIM-45-0-s001.pdf]

WEB APPENDIX FOR "GENERALIZING CONDITIONAL AVERAGE TREATMENT EFFECTS FROM INDIVIDUALS IN RANDOMIZED TRIALS TO ALL TRIAL-ELIGIBLE INDIVIDUALS"

A. Data structure and Identification of the conditional average treatment effect

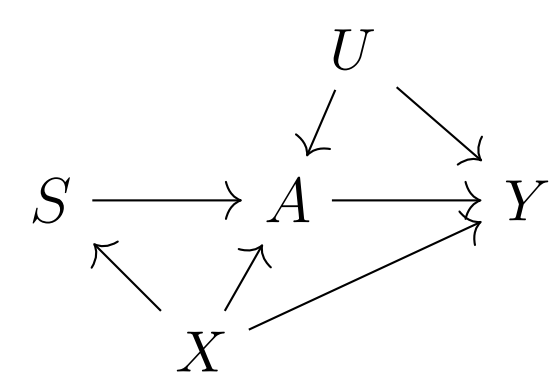

(a) all trial eligible individuals

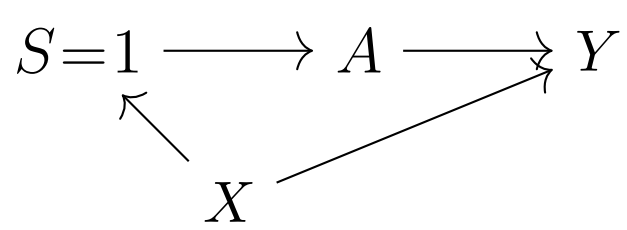

(b) S=1 (randomized individuals; data from trial).

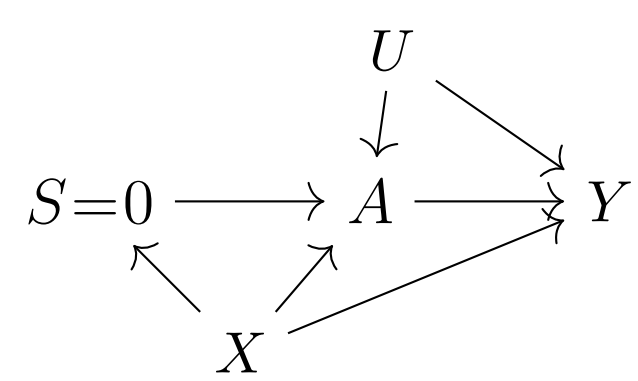

(c) S=0 (non-randomized individuals).

Figure A.1. Directed Acyclic Graphs (DAGs) between randomized and non-randomized individuals: (top) all trial eligible individuals; (bottom left) individuals in S=1; (bottom right) individuals in S=0.

Identification of the conditional average treatment effect (CATE) is given as follows:

$$
\begin{aligned}
E(Y^a|V{=}v) &= \int_x E(Y^a|X{=}x)dF(x|V{=}v) \\
&= \int_x E(Y^a|X{=}x,S{=}1)dF(x|V{=}v) \\
&= \int_x E(Y|A{=}a,X{=}x,S{=}1)dF(x|V{=}v)
\end{aligned}
$$

where the second line follows from the assumption of conditional exchangeability (generalizability) over trial participation S and the last line follows from conditional exchangeability over treatment A in the trial as well as consistency. In the above, note that if we write $X{=}(W,V)$, then under the conditional distribution given $V{=}v$, the value $x{=}(w,\tilde{v})$ with $\tilde{v}\neq v$ has conditional probability (density) zero.

B. Proof of the conditional influence function

As in Chernozhukov et al. (2024), let F_V denote the conditional cumulative distribution function (CDF) of O given V, and define the conditional influence function $\phi_V(a;O)$ to be the *Gateaux derivative* of $\psi(a;V)=\psi(a;F_V)$ with respect to F_V at $\epsilon=0$ as the following:

$$\frac{d\psi(a;F_\epsilon^V)}{d\epsilon}|_{\epsilon=0}=\int \phi_V(a;O)dH_V(o), \quad E[\phi_V(a;O)|V]=0, \tag{B.1}$$

where H_V is a conditional CDF that may be different than F_V, and $F_\epsilon^V=(1-\epsilon)F_V+\epsilon H_V$ for $\epsilon\in[0,1]$. The full derivation is given as follows:

$$\frac{d\psi(a;F_\epsilon^V)}{d\epsilon}|_{\epsilon=0}=\underbrace{\frac{d}{d\epsilon}E\{\gamma_\epsilon(a,X,S=1)|V\}}_{(A)}+\underbrace{\frac{d}{d\epsilon}E_\epsilon\{\gamma(a,X,S=1)|V\}}_{(B)}$$

Term (B) equals the following:

$$\begin{aligned}\frac{d}{d\epsilon}E_\epsilon\{\gamma(a,X,S=1)|V\}&=\int\gamma(a,x,S=1)[H_V(do)-F_V(do)]\\&=\int\{\gamma(a,x,S=1)-\psi(a;V)\}H_V(do)\end{aligned}$$

Term (A) equals the following:

$$\begin{aligned}&\frac{d}{d\epsilon}E\{\gamma_\epsilon(a,X,S=1)|V\}\\&=\frac{d}{d\epsilon}E\left[\underbrace{E_\epsilon(Y|A,X,S)}_{\gamma_\epsilon(Z),\ Z=(A,X,S)}\frac{I(S=1)I(A=a)}{\Pr(S=1|X)f(A|X,S=1)}\middle|V\right]\\&=\frac{d}{d\epsilon}E\left[\gamma_\epsilon(A,X,S)\frac{I(S=1)I(A=a)}{\Pr(S=1|X)f(A|X,S=1)}|V\right]\\&=\frac{d}{d\epsilon}E_\epsilon\left[Y\frac{I(S=1)I(A=a)}{\Pr(S=1|X)f(A|X,S=1)}|V\right]-\frac{d}{d\epsilon}E_\epsilon\left[\gamma(A,X,S)\frac{I(S=1)I(A=a)}{\Pr(S=1|X)f(A|X,S=1)}|V\right]\\&=\int\frac{I(S=1)I(A=a)}{\Pr(S=1|X)f(A|X,S=1)}\{Y-\gamma(A,X,S)\}H_V(do)\end{aligned}$$

C. Proof of Theorem 2

Proof for Theorem 2 is adapted from Chernozhukov et al. (2024). Let $m_h(X,S{=}1,\gamma){=}K_h(V-v)m(X,S{=}1;\gamma)$ and $m_h(X,S{=}1,\hat{\gamma}_{\text{-}l}){=}K_h(V-v)m(X,S{=}1,\hat{\gamma}_{\text{-}l})$; let $\alpha_h(Z){=}K_h(V-v)\alpha(Z)$ and $\hat{\alpha}_h(Z){=}K_h(V-v)\hat{\alpha}(Z)$. Furthermore, let $\xi_h(Z,\alpha_h,\gamma){=}m_h(X,S{=}1,\gamma){+}\alpha_h(Z)(Y-\gamma(A,X,S))$ and $\xi_h(Z_i,\hat{\alpha}_{\text{-}l},\hat{\gamma}_{\text{-}l}){=}m_h(X,S{=}1,\hat{\gamma}_{\text{-}l}){+}\hat{\alpha}_{h,\text{-}l}(Z)(Y-\hat{\gamma}(A,X,S))$.

Differentiating $\hat{\theta}(v){=}\text{argmin}_{\theta,\delta}\sum_{l=1}^{L}\sum_{i\in I_l}\left(\hat{\xi}(Z_i,\hat{\alpha}_{\text{-}l},\hat{\gamma}_{\text{-}l})-\theta-(V_i-v)^T\delta)\right)^2K_h(V_i-v)$ with respect to θ and γ yields:

$$\sum_{l=1}^{L}\sum_{i\in I_l}K_h(V_i-v)\left(\hat{\xi}(Z_i,\hat{\alpha}_{\text{-}l},\hat{\gamma}_{\text{-}l})-\theta-(V_i-v)^\top\delta\right){=}0,\text{ and}$$

$$\sum_{l=1}^{L}\sum_{i\in I_l}(V_i-v)\left(\hat{\xi}(Z_i,\hat{\alpha}_{\text{-}l},\hat{\gamma}_{\text{-}l})-\theta-(V_i-v)^\top\delta\right)K_h(V_i-v){=}0,$$

respectively.

Note that

$$\{\hat{\theta}(v)-\tilde{\theta}(v)\}{=}e_1^T\left(n^{-1}\sum_{l=1}^{L}\sum_{i\in I_l}w_iX_iX_i^T\right)^{-1}\left(n^{-1}\sum_{l=1}^{L}\sum_{i\in I_l}w_iX_i(\hat{\xi}_i-\xi_i)\right),$$

where $e_1{=}(1,0)^T$, $X_i{=}(1,V_i-v)^T$ and $w_i{=}K_h(V_i-v)$.We first aim to show that

$$\frac{1}{n}\sum_{l=1}^{L}\sum_{i\in I_l}\{\xi_h(Z_i,\hat{\alpha}_{\text{-}l},\hat{\gamma}_{\text{-}l})-\xi_h(Z_i,\alpha_h,\gamma)\}{=}o_p\left(\frac{1}{\sqrt{nh}}\right).$$

Let $\phi(z,\alpha,\gamma){=}\alpha(z)\{Y-\gamma(A,X,S{=}1)\}$, $\phi(z,\alpha,\gamma){=}\alpha(z)\{Y-\gamma(A,X,S{=}1)\}$, $\bar{\phi}(\alpha,\gamma){=}P[\phi(Z,\alpha,\gamma)]$ and $\bar{m}_h{=}P[m_h(X,S{=}1,\gamma)]$. First note that:

$$\begin{aligned}&\sqrt{nh}\frac{1}{n}\sum_{l=1}^{L}\sum_{i\in I_l}\{\xi_h(Z_i,\hat{\alpha}_{\text{-}l},\hat{\gamma}_{\text{-}l})-\xi_h(Z_i,\alpha_h,\gamma)\}\\&\quad{=}\frac{\sqrt{nh}}{n}\sum_{l=1}^{L}\sum_{i\in I_l}\{m_h(X_i,S_i{=}1,\hat{\gamma}_{\text{-}l})+\phi(Z_i,\hat{\alpha}_{h,\text{-}l},\hat{\gamma}_{\text{-}l})-m_h(X_i,S_i{=}1,\gamma)-\phi(Z_i,\alpha_h,\gamma)\}\\&\quad{=}\hat{R}_1+\hat{R}_2+\hat{R}_3,\text{ where we define:}\end{aligned}$$

$$\hat{R}_1 = \underbrace{\sqrt{nh}\mathbb{P}_n\Big[m_h(X,S=1,\hat{\gamma}_{\text{-}l}-\gamma)] - \bar{m}_h(\hat{\gamma}_{\text{-}l}-\gamma)\Big]}_{\hat{R}_{11}} +$$
$$\underbrace{\sqrt{nh}\mathbb{P}_n\Big[\phi(Z,\alpha_h,\hat{\gamma}_{\text{-}l}) - \phi(Z,\alpha_h,\gamma) - \bar{\phi}(\alpha_h,\hat{\gamma}_{\text{-}l})\Big]}_{\hat{R}_{12}} +$$
$$\underbrace{\sqrt{nh}\mathbb{P}_n\Big[\phi(Z,\hat{\alpha}_{h,\text{-}l},\gamma) - \phi(Z,\alpha_h,\gamma)\Big]}_{\hat{R}_{13}}$$

$$\begin{aligned}\hat{R}_2 &= \sqrt{nh}\mathbb{P}_n\Big[\phi(Z,\hat{\alpha}_{h,\text{-}l},\hat{\gamma}_{\text{-}l}) - \phi(Z,\alpha_h,\hat{\gamma}_{\text{-}l}) - \phi(Z,\hat{\alpha}_{h,\text{-}l},\gamma) + \phi(Z,\alpha_h,\gamma)\Big] \\ &= \sqrt{nh}\mathbb{P}_n\Big\{\Big[\hat{\alpha}_{h,\text{-}l}(Z) - \alpha_h(Z)\Big]\Big[\gamma - \hat{\gamma}_{\text{-}l}\Big]\Big\}\end{aligned}$$

$$\hat{R}_3 = \sqrt{nh}\Big[\bar{m}_h(\hat{\gamma}_{\text{-}l}-\gamma) + \bar{\phi}(\alpha_h,\hat{\gamma}_{\text{-}l})\Big]$$

where $\mathbb{P}_n$ denotes the empirical measure over i, with slight abuse of notation as $\hat{\gamma}_{-l}$ is computed using fold-specific data. Let $O_{\text{-}l}$ denote observations that do not belong to fold l. We begin with showing that $\hat{R}_1 = o_p(1)$:

$$\begin{aligned}&\sqrt{nh}\mathbb{P}_n\Big[m_h(X,S=1,\hat{\gamma}_{\text{-}l}-\gamma)] - \bar{m}_h(\hat{\gamma}_{\text{-}l}-\gamma)\Big] \\ &\quad = \frac{\sqrt{nh}}{L\sqrt{n_l}}\sum_{l=1}^{L}\Big\{\mathbb{G}_{n_l}\Big[m_h(X,S=1,\hat{\gamma}_{\text{-}l})\Big] - \mathbb{G}_{n_l}\Big[m_h(X,S=1,\gamma)\Big]\Big\} \\ &\quad = \sqrt{\frac{h}{L}}\sum_{l=1}^{L}\Big\{\mathbb{G}_{n_l}\Big[m_h(X,S=1,\hat{\gamma}_{\text{-}l})\Big] - \mathbb{G}_{n_l}\Big[m_h(X,S=1,\gamma)\Big]\Big\}\end{aligned}$$

where $\mathbb{G}_n(X) = \sqrt{n}\{\mathbb{P}_n(X) - P(X)\}$. It then follows that

$$\begin{aligned}H_{n_l,1} &:= \mathbb{G}_{n_l}\Big[m_h(X,S=1,\hat{\gamma}_{\text{-}l})\Big] - \mathbb{G}_{n_l}\Big[m_h(X,S=1,\gamma)\Big] \\ &= \frac{\sqrt{n_l}}{n_l}\sum_{i\in I_l}(\{m_h(X_i,S_i=1,\hat{\gamma}_{\text{-}l}) - m_h(X_i,S_i=1,\gamma)\} - P[m_h(X,S=1,\hat{\gamma}_{\text{-}l}) - m_h(X,S=1,\gamma)])\end{aligned}$$

By independence of observations in l and observations not in l, we have that

$$\begin{aligned}&P(H_{n_l,1}|O_{\text{-}l}) \\ &\quad = \frac{\sqrt{n_l}}{n_l}\sum_{i\in I_l}(P\{m_h(X_i,S_i=1,\hat{\gamma}_{\text{-}l}) - m_h(X_i,S_i=1,\gamma)|O_{\text{-}l}\} - P[m_h(X,S=1,\hat{\gamma}_{\text{-}l}) - m_h(X,S=1,\gamma)]) \\ &\quad = 0\end{aligned}$$

Moreover,

$$\begin{aligned} Var(H_{n_l,1}|O_{\text{-}l}) &= Var[\{m_h(X_i,S{=}1,\hat{\gamma}_{\text{-}l}) - m_h(X_i,S_i{=}1,\gamma)\}|O_{\text{-}l}] \\ &= P\left[\{m_h(X_i,S{=}1,\hat{\gamma}_{\text{-}l}) - m_h(X_i,S_i{=}1,\gamma)\}^2|O_{\text{-}l}\right] \\ &= ||m_h(X,S{=}1,\hat{\gamma}_{\text{-}l}) - m_h(X,S{=}1,\gamma)||^2 \\ &\leq o_p(h^{-1}), \end{aligned}$$

where the last equality holds by our assumptions. Thus $P\left(H^2_{n_l,1}|O_{\text{-}l}\right) = o_p(h^{-1})$. By Chebyshev's inequality,

$$\Pr[|H_{n_l,1}|/c_n > \delta|O_{\text{-}l}] \leq \frac{P\left(H^2_{n_l,1}|O_{\text{-}l}\right)/c_n^2}{\delta^2} = o_p(1)$$

for $c_n = h^{-1/2}$. Since $\Pr(|H_{n_l,1}|/c_n > \delta\ |O_{\text{-}l})$ is a bounded quantity (less than or equal to 1), therefore $H_{n_l,1} = o_p(h^{-1/2})$ and $\hat{R}_{11} = \sqrt{h/L}\sum_{l=1}^{L} H_{n_l,1} = o_p(1)$ (note: $L < \infty$).

Likewise,

$$\begin{aligned} &\sqrt{nh}\mathbb{P}_n\Big[\phi(Z,\alpha_h,\hat{\gamma}_{\text{-}l}) - \phi(Z,\alpha_h,\gamma) - \bar{\phi}(\alpha_h,\hat{\gamma}_{\text{-}l})\Big] \\ &\quad= \sqrt{nh}\mathbb{P}_n\Big[\phi(Z,\alpha_h,\hat{\gamma}_{\text{-}l}) - \phi(Z,\alpha_h,\gamma) - \bar{\phi}(\alpha_h,\hat{\gamma}_{\text{-}l}) + \bar{\phi}(\alpha_h,\gamma)\Big] \quad \text{since } \bar{\phi}(\alpha_h,\gamma) = 0 \\ &\quad= \sqrt{nh}\mathbb{P}_n\Big[\phi(Z,\alpha_h,\hat{\gamma}_{\text{-}l} - \gamma) - \bar{\phi}(\alpha_h,\hat{\gamma}_{\text{-}l} - \gamma)\Big] \\ &\quad= \sqrt{\frac{h}{L}}\sum_{l=1}^{L}\underbrace{\Big\{\mathbb{G}_{n_l}\Big[\phi(Z,\alpha_h,\hat{\gamma}_{\text{-}l})\Big] - \mathbb{G}_{n_l}\Big[\phi(Z,\alpha_h,\gamma)\Big]\Big\}}_{H_{n_l,2}}, \quad \text{and similarly} \end{aligned}$$

$$\begin{aligned} &\sqrt{nh}\mathbb{P}_n\Big[\phi(Z,\hat{\alpha}_{h,\text{-}l},\gamma) - \phi(Z,\alpha_h,\gamma)\Big] \\ &\quad= \sqrt{nh}\mathbb{P}_n\Big[\phi(Z,\hat{\alpha}_{h,\text{-}l} - \alpha_h,\gamma) - \bar{\phi}(\hat{\alpha}_{h,\text{-}l} - \alpha_h,\gamma)\Big] \\ &\quad= \sqrt{\frac{h}{L}}\sum_{l=1}^{L}\underbrace{\Big\{\mathbb{G}_{n_l}\Big[\phi(Z,\hat{\alpha}_{h,\text{-}l},\gamma)\Big] - \mathbb{G}_{n_l}\Big[\phi(Z,\alpha_h,\gamma)\Big]\Big\}}_{H_{n_l,3}}. \end{aligned}$$

Analogous to the argument for $H_{n_l,1}$, it follows that $\hat{R}_{12} = o_p(1)$ and that $\hat{R}_{13} = o_p(1)$.

Next, we consider $\hat{R}_2$, which equals:

$$\hat{R}_2 = \sqrt{nh}\mathbb{P}_n\Big\{\Big[\hat{\alpha}_{h,\text{-}l}(Z) - \alpha_h(Z)\Big]\Big[\gamma - \hat{\gamma}_{\text{-}l}\Big]\Big\}$$

$$=\frac{\sqrt{nh}}{L}\sum_{l=1}^{L}\underbrace{\frac{1}{n_l}\sum_{i\in I_l}\Big[\hat{\alpha}_{h,\text{-}l}(Z_i)-\alpha_h(Z_i)\Big]\Big[\gamma-\hat{\gamma}_{\text{-}l}\Big]}_{\hat{R}_{2l}}$$

Immediately by our assumption, it follows that:

$$\begin{aligned}
P\Big(|\hat{R}_{2l}|\ \Big|O_{\text{-}l}\Big)=&P\left\{\frac{1}{n_l}\left|\sum_{i\in I_l}\Big[\hat{\alpha}_{h,\text{-}l}(Z_i)-\alpha_h(Z_i)\Big]\Big[\gamma-\hat{\gamma}_{\text{-}l}\Big]\right|\Big|O_{\text{-}l}\right\}\\
\leq&\frac{1}{n_l}\sum_{i\in I_l}\left|P\Big\{\Big[\hat{\alpha}_{h,\text{-}l}(Z_i)-\alpha_h(Z_i)\Big]\Big[\gamma-\hat{\gamma}_{\text{-}l}\Big]\Big|O_{\text{-}l}\Big\}\right|\ \text{ (by triangle inequality)}\\
\leq&\frac{1}{n_l}\sum_{i\in I_l}||\hat{\alpha}_{h,\text{-}l}(Z_i)-\alpha_h(Z_i)||\ ||\gamma-\hat{\gamma}_{\text{-}l}||\ \text{ (by Cauchy Schwartz)}\\
\leq&Ch^{-1}o_p(\sqrt{h/n})\ \text{ (by assumption 1.III)}
\end{aligned}$$

By Markov's inequality:

$$\Pr\Big(|\hat{R}_{2l}|>\varepsilon/\sqrt{nh}\Big|O_{\text{-}l}\Big)\leq\frac{P\Big(|\hat{R}_{2l}|\Big|O_{\text{-}l}\Big)}{\varepsilon/\sqrt{nh}}=o_p(1).$$

Since $\Pr\Big(|\hat{R}_{2l}|>\varepsilon/\sqrt{nh}\Big|O_{\text{-}l}\Big)$ is a bounded quantity (less than or equal to 1), it follows that $\Pr(|\hat{R}_{2l}|>\varepsilon/\sqrt{nh})\to 0$ as $n\to\infty$, and as such $\hat{R}_{2l}=o_p(1/\sqrt{nh})$.

Therefore, we have that:

$$\begin{aligned}
|\hat{R}_2|\leq&\frac{\sqrt{nh}}{L}\sum_{l=1}^{L}\Big(|\hat{R}_{2l}|\Big)\\
=&\frac{\sqrt{nh}}{L}L\cdot o_p(1/\sqrt{nh})=o_p(1).
\end{aligned}$$

Finally, consider $\hat{R}_3=\sqrt{nh}\Big[\bar{m}_h(\hat{\gamma}_{\text{-}l}-\gamma)+\bar{\phi}(\alpha_h,\hat{\gamma}_{\text{-}l})\Big]$. For notational brevity, let $\gamma_a=\gamma(a,X,S=1)$ and let $\hat{\gamma}_{\text{-}l,a}=\hat{\gamma}_{\text{-}l}(a,X,S=1)$, for $a=0,1$. Similarly, let $\gamma_A=\gamma(A,X,S=1)$ and let $\hat{\gamma}_{\text{-}l,A}=\hat{\gamma}_{\text{-}l}(A,X,S=1)$.

$$\begin{aligned}
\hat{R}_3=&\sqrt{nh}\int K_h(V-v)[(\hat{\gamma}_{\text{-}l,1}-\gamma_1)-(\hat{\gamma}_{\text{-}l,0}-\gamma)]F(dx)+\\
&\sqrt{nh}\int K_h(V-v)\frac{S\cdot(2A-1)}{\Pr(S=1|X)f(A|X,S=1)}[Y-\hat{\gamma}_{\text{-}l}(A,X,S=1)]F(do)
\end{aligned}$$

$$=\sqrt{nh}\int K_h(V-v)(\hat{\gamma}_{\text{-}l,1}-\gamma_1)F(dx)-\int K_h(V-v)(\hat{\gamma}_{\text{-}l,0}-\gamma)F(dx)+$$
$$\sqrt{nh}\int K_h(V-v)\frac{S\cdot(2A-1)}{\Pr(S=1|X)f(A|X,S=1)}[\gamma_A-\hat{\gamma}_{\text{-}l,A}]F(do)$$
$$=\sqrt{nh}P\left[K_h(V-v)(\hat{\gamma}_{\text{-}l,1}-\gamma_1)\frac{S\cdot A}{\Pr(S=1|X)\Pr(A=1|X,S=1)}\right]-$$
$$\sqrt{nh}P\left[K_h(V-v)(\hat{\gamma}_{\text{-}l,0}-\gamma)\frac{S\cdot(1-A)}{\Pr(S=1|X)\Pr(A=0|X,S=1)}\right]+$$
$$\sqrt{nh}P\left[K_h(V-v)\frac{S\cdot(2A-1)}{\Pr(S=1|X)f(A|X,S=1)}[\gamma_A-\hat{\gamma}_{\text{-}l}(A,X,S=1)]\right]$$
$$=-\sqrt{nh}P\left[K_h(V-v)\frac{S\cdot(2A-1)}{\Pr(S=1|X)f(A|X,S=1)}[\gamma_A-\hat{\gamma}_{\text{-}l,A}]\right]+$$
$$\sqrt{nh}P\left[K_h(V-v)\frac{S\cdot(2A-1)}{\Pr(S=1|X)f(A|X,S=1)}[\gamma_A-\hat{\gamma}_{\text{-}l,A}]\right]=0$$

The proof is complete by repeating the same arguments above and replacing all instances of $K_h(V-v)$ with $K_h(V-v)(V-v)$ such that it can similarly be argued that $\frac{1}{n}\sum_{l=1}^{L}\sum_{i\in I_l}K_h(V_i-v)(V_i-v)\{\xi(Z_i,\hat{\alpha}_{\text{-}l},\hat{\gamma}_{\text{-}l})-\xi(Z_i,\alpha_h,\hat{\gamma})\}=o_p\left(\frac{1}{\sqrt{nh}}\right)$.

C.1 Derivation of $\sigma^2(v)=E\{\xi(Z;m,\alpha)|V=v\}$ Here, we show that $\sigma^2(v)=Var\{\xi(Z;m,\alpha)|V=v\}$ equals:

$$E\left[\frac{1}{Pr(S=1|X)f(v)}\left\{\frac{\rho_1(X)}{\Pr(A=1|X,S=1)}-\frac{\rho_0(X)}{\Pr(A=0|X,S=1)}\right\}+\frac{1}{f(v)}\{m(X,S=1)-\theta(v)\}\right]$$

where $\rho_a(X):=Var(Y|A=a,X,S=1)$ for $a=0,1$.

Proof.

$$Var\{\xi(Z;m,\alpha)|V=v\}=E\left[\{\xi(Z;m,\alpha)-E(\xi(Z;m,\alpha)|V=v)\}^2|V=v\right]$$
$$=E\left[\{\xi(Z;m,\alpha)-\theta(v)\}^2|V=v\right]$$
$$=E\left[\left\{\frac{S\cdot(2A-1)}{\Pr(S=1|X)f(A|X,S=1)}\{Y-\gamma(Z)\}-m(X,S=1)-\theta(v)\right\}^2|V=v\right]$$
$$=E\left[\frac{S(2A-1)^2}{\Pr(S=1|X)f(A|X,S=1)}Var(Y|A,X,S=1)|V=v\right]+\underbrace{E\left[\{m(X,S=1)-\theta(v)\}^2|V=v\right]}_{(*)}$$
$$=E\left[\frac{1}{\Pr(S=1|X)}\left\{\frac{Var(Y|A=1,X,S=1)}{\Pr(A=1|X,S=1)}+\frac{Var(Y|A=0,X,S=1)}{\Pr(A=0|X,S=1)}\right\}|V=v\right]+(*)$$

$$=E\left[\frac{1}{\Pr(S=1|X)}\left\{\frac{Var(Y|A=1,X,S=1)}{\Pr(A=1|X,S=1)}+\frac{Var(Y|A=0,X,S=1)}{\Pr(A=0|X,S=1)}\right\}+\{m(X,S=1)-\theta(v)\}^2|V=v\right]$$

□

C.2 Influence function for $\beta=(\theta,\delta)^T$. For ease of notation, let $\beta=(\theta,\delta)^T$. The influence function in the nonparametric model $\mathcal{M}_{np}$ is defined as the unique mean zero, finite variance random variable $\varphi^{\text{eff}}(O)$ such that

$$\frac{d\beta(F_\epsilon)}{d\epsilon}\Big|_{\epsilon=0}=E\{\varphi^{\text{eff}}(O)S(O)\}$$

where $d\mu^g(F_\epsilon)/d\epsilon|_{\epsilon=0}$ is known as the pathwise derivative of parameter μ^g along a parametric submodel indexed by ϵ, and $S(O)$ is the score function of the parametric submodel evaluated at $\epsilon=0$ (such that $F_{\epsilon=0}=F$). For ease of notation, we also let $\beta:=\beta(F)$, $K_{hv}(V):=K_h(V-v)$, $g(V)=(1,V-v)^T$. Since $V\subset X$, we let $X=(W,V)$. We will denote nuisance functions by η and the corresponding nuisance function estimates as $\hat{\eta}$ and let

$$\xi(O;\eta):=\frac{S\cdot(2A-1)}{\Pr(S=1|X)f(A|X,S=1)}\{Y-\gamma(Z)\}+m(X,S=1;\gamma)$$

and

$$\phi(O;\eta,\beta):=g(V)K_{hv}(V)\left[\xi(Z;\eta)-g(V)^T\beta\right].$$

The influence function of β is given as follows:

$$\varphi(O)=\mathbb{D}^{-1}\left[g(V)K_{hv}(V)\left\{\xi(Z;\eta)-g(V)^T\beta\right\}\right],$$

where $\mathbb{D}=E[g(V)K_{hv}(V)g(V)^T]$.

We derive an expression for $\frac{d\beta(F_\epsilon)}{d\epsilon}\Big|_{\epsilon=0}$. First note that:

$$\beta=\underset{\tilde{\beta}}{\arg\min}\,E[K_{hv}(V)\{\theta(V)-g(V)^T\tilde{\beta}\}^2],$$

where $g(V)^T\beta$ is equal to the smoothed version of θ, θ^*. Moreover,

$$E\left[g(V)K_{hv}(V)\{\theta(V)-g(V)^T\beta\}\right]=0.$$

Differentiating with respect to ϵ and evaluating at $\epsilon=0$ yields:

$$\frac{d\beta(F_\epsilon)}{d\epsilon}\Big|_{\epsilon=0}=\mathbb{D}^{-1}\int_v g(v)K_{hv}(v)\Big[\theta'(v;0)+\left\{\theta(v;0)-g(v)^T\beta(F)\right\}S(v)\Big]f(v)d\mu(v)$$

where $\theta(v;\epsilon)=\int_w m(w,v,S=1,\gamma;\epsilon)f(w|v;\epsilon)d\mu(w)$ with $\theta(v;0)=\theta(v)$, and $\theta'(v;\epsilon)=\frac{d\theta(v;\epsilon)}{d\epsilon}$. It can be shown that

$$\begin{aligned}\theta'(v;0)=&E[E\{YS(Y|A=1,X,S=1)|A=1,X,S=1\}|V=v]-\\&E[E\{YS(Y|A=0,X,S=1)|A=0,X,S=1\}|V=v]+\\&E[m(X,S=1;\gamma)S(W|V)|V=v].\end{aligned}$$

Therefore it follows that

$$\begin{aligned}\frac{d\beta(F_\epsilon)}{d\epsilon}\Big|_{\epsilon=0}=&\mathbb{D}^{-1}\int_v g(v)K_{hv}(v)\Big[\theta'(v,0)+\big\{\theta(v,0)-g(v)^T\beta(F)\big\}S(v)\Big]f(v)d\mu(v)\\=&\mathbb{D}^{-1}\int_v g(v)K_{hv}(v)\Bigg(E[E\{YS(Y|A=1,X,S=1)|A=1,X,S=1\}|V=v]-\\&E[E\{YS(Y|A=0,X,S=1)|A=0,X,S=1\}|V=v]+\\&E[m(X,S=1;\gamma)S(W|V)|V=v]+\\&\big\{\theta(v)-g(v)^T\beta(F)\big\}S(v)\Bigg)f(v)d\mu(v)\end{aligned}$$

To illustrate the influence function satisfies the above, we first consider $\mathbb{D}^{-1}\left[g(V)K_{hv}(V)\left\{\xi(Z;\eta)-g(V)^T\beta\right\}\right]S(Y|Z)$, where as before $Z=(A,X,S)$:

$$\begin{aligned}&\mathbb{D}^{-1}E\left[g(V)K_{hv}(V)\left\{\xi(Z;\eta)-g(V)^T\beta\right\}\right]S(Y|Z)]\\&=\mathbb{D}^{-1}E\left[g(v)K_{hv}(V)]\frac{S(2A-1)}{\Pr(S=1|X)f(A|X,S=1)}YS(Y|Z)\right]\\&=\mathbb{D}^{-1}E\Big(g(v)K_{hv}(V)]\Big[E\{YS(Y|A=1,X,S=1)|A=1,X,S=1\}-\\&\qquad E\{YS(Y|A=0,X,S=1)|A=0,X,S=1\}\Big]\Big)\\&=\mathbb{D}^{-1}\int_v g(v)K_{hv}(v)\Big(E[E\{YS(Y|A=1,X,S=1)|A=1,X,S=1\}|V=v]-\\&\qquad E[E\{YS(Y|A=0,X,S=1)|A=0,X,S=1\}|V=v]\Big)f(v)d\mu(v)\end{aligned}$$

Next, we consider $\mathbb{D}^{-1}\left[g(V)K_{hv}(V)\left\{\xi(Z;\eta)-g(V)^T\beta\right\}\right]S(Z)$:

$$\begin{aligned}&\mathbb{D}^{-1}E\left[g(V)K_{hv}(V)\left\{\xi(Z;\eta)-g(V)^T\beta(F)\right\}\right]S(Z)]\\&=\mathbb{D}^{-1}\Big(E\Big[g(V)K_{hv}(V)E\{m(X,S=1;\gamma)S(W|V)|V\}\Big]+\\&\qquad E\Big[g(V)K_{hv}(V)m(X,S=1;\gamma)S(V)\Big]\end{aligned}$$

$$E\Big[g(V)K_{hv}(V)g(V)^T\beta(F)S(V)\Big]\Big)$$

$$=\mathbb{D}^{-1}\int_v g(v)K_{hv}(v)\Big(E[m(X,S{=}1;\gamma)S(W|V)|V{=}v]+$$

$$\big\{\theta(v)-g(v)^T\beta(F)\big\}S(v)\Big)f(v)d\mu(v)$$

where the last equality is realized by noting that $E[m(X,S{=}1;\gamma)|V{=}v]{=}\theta(v)$.

C.3 Asymptotic Normality of $\hat{\beta}$ Formally, we assume the following conditions throughout:

C1. $\hat{\beta}-\beta^*{=}o_p(1)$, $\eta(\hat{F})-\eta(F){=}o_p(1)$.

C2. $\mathbb{D}{:=}E_P\left[g(V)K_{hv}(V)g(V)^T\right]$ is nonsingular.

C3. $E_P[\phi(O;\eta,\beta^*)^2]{<}\infty$

Under the aforementioned conditions, and further suppose that the product mean-squared error condition of assumption 1 holds, then it follows that:

$$\sqrt{n}\left(\hat{\beta}-\beta^*\right)\rightsquigarrow MVN\left(0,\mathbb{D}^{-1}\mathrm{Cov}\{\phi(O;\eta,\beta)\}\mathbb{D}^{-1^T}\right),$$

where β^* denotes the smoothed version of β, and as before, let

$$\phi(O;\eta,\beta){:=}g(V)K_{hv}(V)\left[\xi(Z;\eta)-g(V)^T\beta\right].$$

Proof. By construction, we solve for β in the following estimating equation:

$$\mathbb{P}_n[\phi(O;\hat{\eta};\beta)]{=}0.$$

Suppose that $\hat{\beta}{:=}\beta(\hat{F})$ solves the aforementioned estimating equation. Thus, by expanding the estimating equation around $\beta{:=}\beta(F)$ (with a slight abuse of notation), we obtain the following:

$$\mathbb{P}_n\Big[\phi(O;\hat{\eta};\hat{\beta})\Big]{=}\mathbb{P}_n[\phi(O;\hat{\eta};\beta)]+\mathbb{P}_n\Big[\phi'(O;\hat{\eta};\tilde{\beta})\Big](\hat{\beta}-\beta^*)$$

where $\tilde{\beta}$ is an intermediate value between $\hat{\beta}$ and β^*, and $\phi'(O;\hat{\eta};\tilde{\beta}){=}\frac{d\phi(O;\hat{\eta};\tilde{\beta})}{d\beta}$. After rearranging the terms in the previous and multiplying the left-hand-side and right-hand-side by $\sqrt{n}$, we obtain:

$$\sqrt{n}(\hat{\beta}-\beta^*){=}-\left(\mathbb{P}_n\Big[\phi'(O;\hat{\eta};\tilde{\beta})\Big]\right)^{-1}\sqrt{n}\mathbb{P}_n[\phi(O;\hat{\eta};\beta^*)] \qquad \text{(C.2)}$$

Note that by the Uniform Law of Large Numbers and by the Continuous Mapping Theorem, $\mathbb{P}_n\left[\phi'(O;\hat{\eta};\tilde{\beta})\right]\xrightarrow{p}\mathbb{D}$, provided that condition C2. holds. We will utilize this result later.

Next, we look at the term $\sqrt{n}\mathbb{P}_n[\phi(O;\hat{\eta};\beta^*)]$ in (C.2):

$$\begin{aligned}\sqrt{n}\mathbb{P}_n[\phi(O;\hat{\eta};\beta^*)] &= \sqrt{n}(\mathbb{P}_n[\phi(O;\hat{\eta};\beta^*)]-\mathbb{P}[\phi(O;\eta;\beta^*)])\\ &= \underbrace{\mathbb{G}_n[\phi(O;\eta,\beta^*)]}_{T_1}+\underbrace{\mathbb{G}_n[\phi(O;\hat{\eta},\beta^*)-\phi(O;\eta,\beta^*)]}_{T_2}+\sqrt{n}\underbrace{P[\phi(O;\hat{\eta},\beta^*)-\phi(O;\eta,\beta^*)]}_{R}\end{aligned}$$

The first term given by T_1 is a centered sample average which, under conditions C3. converges to a mean zero Multivariate Normal distribution by the central limit theorem such that $\mathbb{G}_n[\phi(O;\eta,\beta)]\rightsquigarrow MVN(0,\text{Cov}\{\phi(O;\eta,\beta)\})$. The second term T_2 is an empirical process term, which can be shown to be $o_p(1)$ under sample splitting and cross fitting to overcome issues with overfitting, analogous to the proof provided in C (Chernozhukov et al., 2018; Wen et al., 2023). Then the results follow once we show that the remainder term $\sqrt{n}R$ is $o_p(1)$. This can be accomplished, for example, if the nuisance functions are consistently estimated at a rate faster than $n^{-1/4}$. To see this, we note that

$$\sqrt{n}R\leq\sqrt{n}\sup_V\Big(g(V)K_{hv}(V)\Big)P\Big[\{\hat{\alpha}(Z)-\alpha(Z)\}\{\gamma(A,X,S=1)-\hat{\gamma}(A,X,S=1)\}\Big].$$

Thus, putting it all together, it follows then in:

$$\sqrt{n}(\hat{\beta}-\beta^*)=-\left(\mathbb{P}_n\left[\phi'(O;\hat{\eta};\tilde{\beta})\right]\right)^{-1}\sqrt{n}\mathbb{P}_n[\phi(O;\hat{\eta};\beta^*)]$$

we have that $\mathbb{P}_n\left[\phi'(O;\hat{\eta};\tilde{\beta})\right]\xrightarrow{p}\mathbb{D}$, and

$$\sqrt{n}\mathbb{P}_n[\phi(O;\hat{\eta};\beta^*)]\rightsquigarrow MVN(0,\text{Cov}\{\phi(O;\eta,\beta^*)\}).$$

By Slutsky's theorem, it follows then:

$$\sqrt{n}\left(\hat{\beta}-\beta^*\right)\rightsquigarrow MVN\left(0,\mathbb{D}^{-1}\text{Cov}\{\phi(O;\eta,\beta^*)\}D^{-1^T}\right).$$

In particular for $k=0,\ldots,p$,

$$\sqrt{n}\left(\hat{\beta}_k-\beta^*_k\right)\rightsquigarrow N\left(0,\left[\mathbb{D}^{-1}\text{Cov}\{\phi(O;\eta,\beta^*)\}\mathbb{D}^{-1^T}\right]_{kk}\right),$$

where $\left[\mathbb{D}^{-1}\text{Cov}\{\phi(O;\eta,\beta^*)\}\mathbb{D}^{-1^T}\right]_{kk}$ denotes the kth diagonal element of the variance-covariance matrix, and $\text{Cov}\{\phi(O;\eta,\beta^*)\}=E\{\phi(O;\eta,\beta^*)\phi(O;\eta,\beta^*)^T\}$. □

D. Simulation details

D.1 Simulation details described in the main text. We simulate cohorts of n=(2500, 5000) individuals. In each cohort we vary the number of randomized participants, $n_{s=1}$, across three scenarios $n_{s=1}$=(125, 500, 1000).

Following Dahabreh et al. (2019), the data generation process was conducted as follows: generation of covariates from a cohort; selection for trial participation; (random) treatment assignment amongst trial participants; generation of outcome of interest Y. Specifically, for binary outcome Y, data were simulated as follows:

(1) *Covariates*: simulate measured covariates from a cohort. In this simulation, we generate X=(X_1,X_2,X_3) where $X_1 \sim$Ber(0.5), $X_2 \sim$Unif(0,4) and $X_3 \sim$ N(0,1).
(2) *Selection for trial participation:* We "select" observations for trial participation using a logistic-linear model such that the probability of being selected into the trial is given by $\Pr(S{=}1|X){=}\text{expit}(\beta_0{+}X_1{+}X_2{+}X_3)$, where the intercept β_0 is determined numerically to achieve the desired average sample size $n_{s=1}$=(125, 500, 1000).
(3) *Treatment in trial:* We generate an indicator of unconditionally randomized A assignment among randomized individuals. The A assignment mechanism is given by $A{\sim}\text{Ber}(\Pr(A{=}1|S{=}1))$ where $\Pr(A{=}1|S{=}1){=}0.5$.
(4) *Outcomes in trial:* We generate outcomes in S=1 using $Y{\sim}\text{Ber}(\Pr(Y{=}1|A,X))$ where $\Pr(Y{=}1|A,X){=}{-}4{+}3A{+}X_1{+}1.5X_2{+}X_3{-}0.5X_2^2A$.

For continuous outcome Y, data were simulated as follows:

(1) *Covariates*: simulate measured covariates from a cohort. In this simulation, we generate X=(X_1,X_2,X_3) where $X_1 \sim$Ber(0.5), $X_2 \sim$Unif(0,4) and $X_3 \sim$ N(0,1).
(2) *Selection for trial participation:* We "select" observations for trial participation using a logistic-linear model such that the probability of being selected into the trial is given by $\Pr(S{=}1|X){=}\text{expit}(\beta_0{+}X_1{+}X_2{+}X_3)$, where the intercept β_0 is determined numerically to achieve the desired average sample size $n_{s=1}$=(125, 500, 1000).
(3) *Treatment in trial:* We generate an indicator of unconditionally randomized A assignment among randomized individuals. The A assignment mechanism is given by $A{\sim}\text{Ber}(\Pr(A{=}1|S{=}1))$ where $\Pr(A{=}1|S{=}1){=}0.5$.
(4) *Outcomes in trial:* We generate outcomes in S=1 using $Y{\sim}\text{N}(\mu,1)$ where $\mu{=}10{+}2A{+}X_1{+}X_2{+}X_3{+}(0.2X_2^2{-}0.1X_2^3)A$.

For the continuous outcome, the simulation design described above, we know the true functional form of the CATE which is given by $2+0.2X_2^2-0.1X_2^3$. Results of for the continuous outcome is provided in Table D.1 and Figure D.2.

As mentioned in the main manuscript, for the continuous outcome setting, we additionally consider an oracle estimator that uses the same first-stage CATE estimates but replaces the second-stage model with the true functional form.

D.1.1 Results for continuous outcome

Method	Integrated |Bias|	Integrated RMSE	Integrated CP
	$n_{s=1}$=125, n=2500		
Local linear regression	0.110	1.374	90
Linear regression (naive)	0.367	1.411	85
Linear regression (oracle)	0.093	1.434	94
	$n_{s=1}$=500, n=2500		
Local linear regression	0.082	0.571	91
Linear regression (naive)	0.366	0.646	60
Linear regression (oracle)	0.058	0.518	93
	$n_{s=1}$=1000, n=2500		
Local linear regression	0.064	0.289	88
Linear regression (naive)	0.366	0.451	40
Linear regression (oracle)	0.014	0.228	94
	$n_{s=1}$=125, n=5000		
Local linear regression	0.074	1.413	90
Linear regression (naive)	0.362	1.356	85
Linear regression (oracle)	0.127	1.417	93
	$n_{s=1}$=500, n=5000		
Local linear regression	0.056	0.759	91
Linear regression (naive)	0.365	0.797	63
Linear regression (oracle)	0.033	0.691	92
	$n_{s=1}$=1000, n=5000		
Local linear regression	0.058	0.415	90
Linear regression (naive)	0.366	0.600	49
Linear regression (oracle)	0.026	0.429	94

TABLE D.1. Integrated Bias, integrated RMSE, and integrated 95% Coverage Probability (CP) for continuous outcome. For the $n_{s=1}$=125 scenarios, the most extreme 5% of values were trimmed.

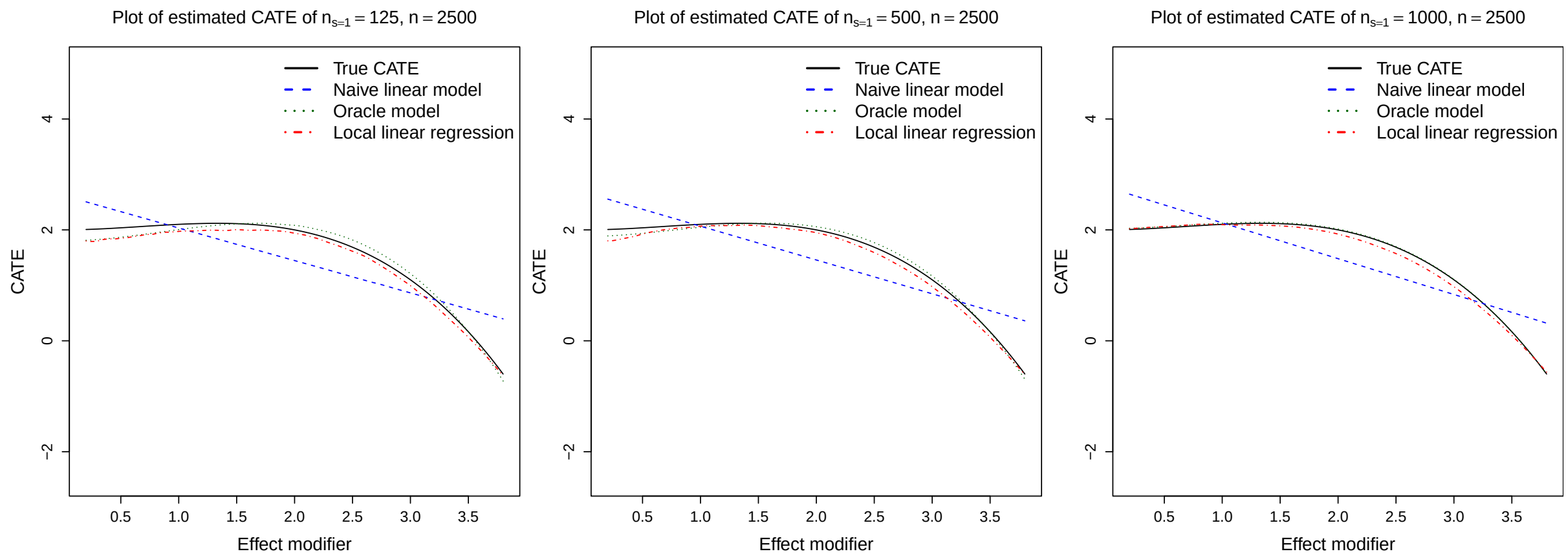

FIGURE D.2. Comparison of proposed estimator with naive and oracle estimators across trial participation size $n_{s=1}$=(125, 500, 1000) for n= 2500 for *continuous outcome*. 95% coverage probability across values of effect modifier (X_2) is shown for the proposed estimator.

D.1.2 A comparison with an estimator based solely on trial data. In each simulated dataset, we also obtained a trial only estimate of the treatment effect using the DR-learner of (Kennedy, 2023) where the second-stage regression is performed via local linear regression.

In the continuous setting, X_2 is the only effect modifier and satisfies $E(Y^{a=1}-Y^{a=0}|X)=2+0.2X_2^2-0.1X_2^3$, and so the conditional treatment effect depends only on X_2 and does not depend on X_1, X_3 or S. As a result,

$$E(Y^{a=1}-Y^{a=0}|X)=E(Y^{a=1}-Y^{a=0}|X_2)$$

and $E(Y^{a=1}-Y^{a=0}|X)=E(Y^{a=1}-Y^{a=0}|X,S=1)=E(Y^{a=1}-Y^{a=0}|X_2,S=1)$. Thus,

$$E(Y^{a=1}-Y^{a=0}|X_2)=E(Y^{a=1}-Y^{a=0}|X_2,S=1)$$

and trial only estimation is valid under this setting (see Table D.3; results are comparable to those of the proposed method in Table D.1). In the binary setting, X_2 remains the only effect modifier, but on the logit scale. Specifically,

$$\text{logit}[E(Y^{a=1}|X)]-\text{logit}[E(Y^{a=0}|X)]=3-0.5X_2^2,$$

which does not depend on (X_1,X_3). However, because the logit link is non-collapsible, the fact that $\text{logit}[E(Y^{a=1}|X)]-\text{logit}[E(Y^{a=0}|X)]$ does not depend on (X_1,X_3) on the logit scale does not imply that $E(Y^{a=1}-Y^{a=0}|X)$ does not depend on (X_1,X_3) in the risk difference scale. To see this,

$$\begin{aligned}E(Y^{a=1}-Y^{a=0}|X)&=E(Y^{a=1}-Y^{a=0}|X,S=1)\\&=\text{expit}[g_1(X_1,X_2,X_3)]-\text{expit}[g_2(X_1,X_2,X_3)],\end{aligned}$$

where $g_1(X)$ and $g_2(X)$ are some arbitrary functions of $X=(X_1,X_2,X_3)$. As a result, it does not necessarily follow that $E(Y^{a=1}-Y^{a=0}|X_2,S=1)=E(Y^{a=1}-Y^{a=0}|X_2)$ since in general,

$$p(x_1,x_3|x_2,S=1)\neq p(x_1,x_3|x_2).$$

Figure D.3 illustrates that the differences between true trial only CATE and true CATE among the trial-eligible individuals, and Table D.2 shows the performance of the aforementioned trial only estimator for binary outcomes, which shows some bias and under-coverage of the 95% confidence intervals.

Next, we consider more general settings where there are multiple effect modifiers but CATE only considers a subset of the effect modifiers. In these cases, trial only estimators give very biased results for the CATE among the trial eligible individuals.

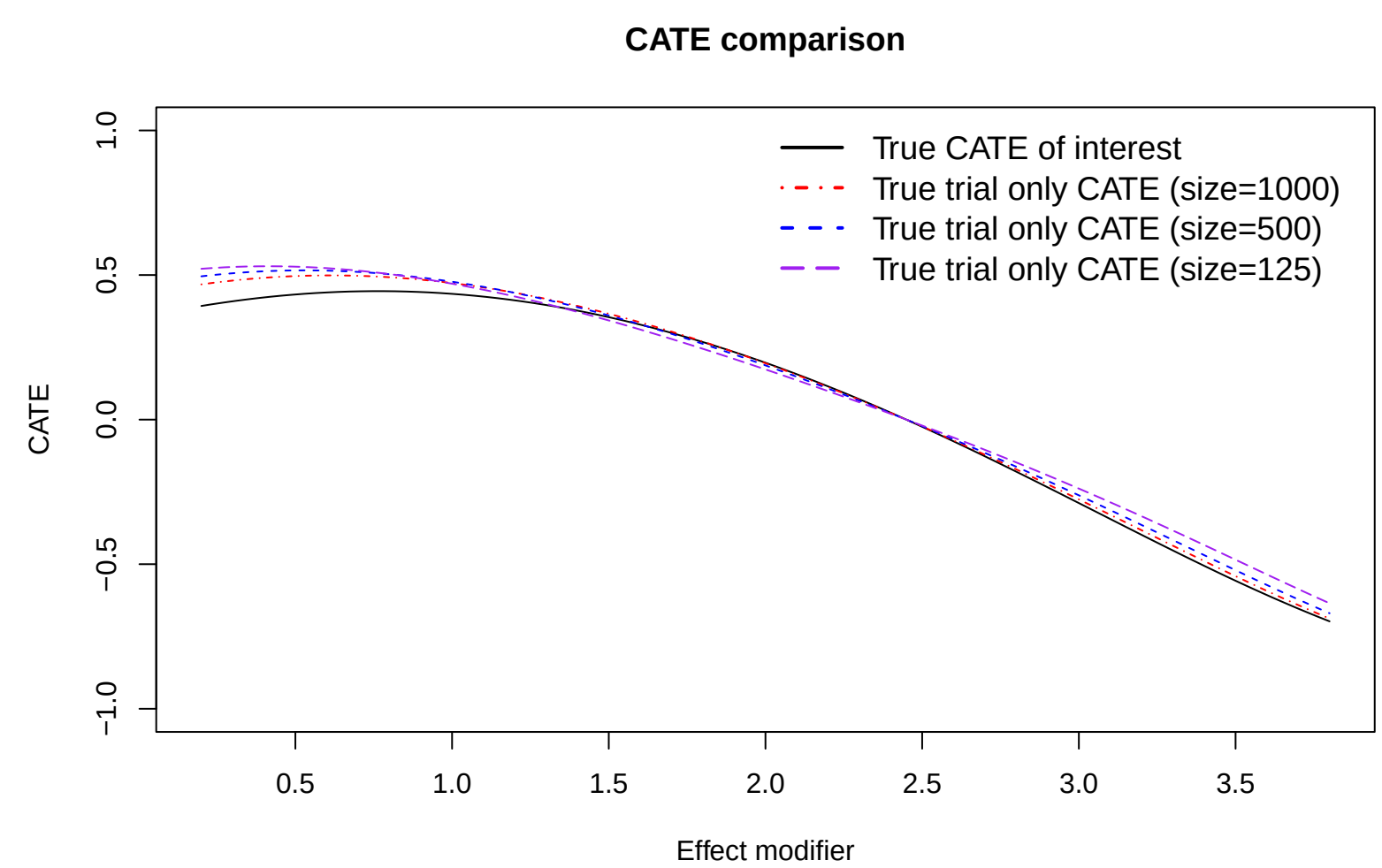

Figure D.3. A comparison of true CATE of interest among all trial-eligible individuals versus true CATE of trial only participants.

	Integrated |Bias|	Integrated RMSE	Integrated CP
	$n_{s=1}=125$		
Local linear (trial only)	0.072	0.189	88
	$n_{s=1}=500$		
Local linear (trial only)	0.051	0.111	82
	$n_{s=1}=1000$		
Local linear (trial only)	0.042	0.081	82

Table D.2. Results from the local linear regression estimator based on trial only participants for binary outcome.

	Integrated |Bias|	Integrated RMSE	Integrated CP
	$n_{s=1}$=125		
Local linear (trial only)	0.135	0.669	83
	$n_{s=1}$=500		
Local linear (trial only)	0.059	0.282	86
	$n_{s=1}$=1000		
Local linear (trial only)	0.047	0.174	88

TABLE D.3. Results from the local linear regression estimator based on trial only participants for continuous outcome.

D.2 Additional simulation studies: comparison with an estimator based solely on trial data.

D.2.1 Binary outcomes. We consider the same data generating mechanism for binary outcomes as Subsection D.1, with the exception that outcomes for trial participants are generated according to a Bernoulli model: $Y \sim \text{Ber}(\Pr(Y=1|A,X))$ where $\Pr(Y=1|A,X) = -2+A-X_1+X_2+X_3+X_2^2A-2X_3A$.

There are two effect modifiers (on the logit scale): (X_2, X_3). As show in Figure D.4, difference between the true CATE in the trial eligible individuals differ greatly than the CATE in the trial participants. This difference is much noticeable compared with the setting where X_2 was the only effect modifier on the logit scale (Figure D.3).

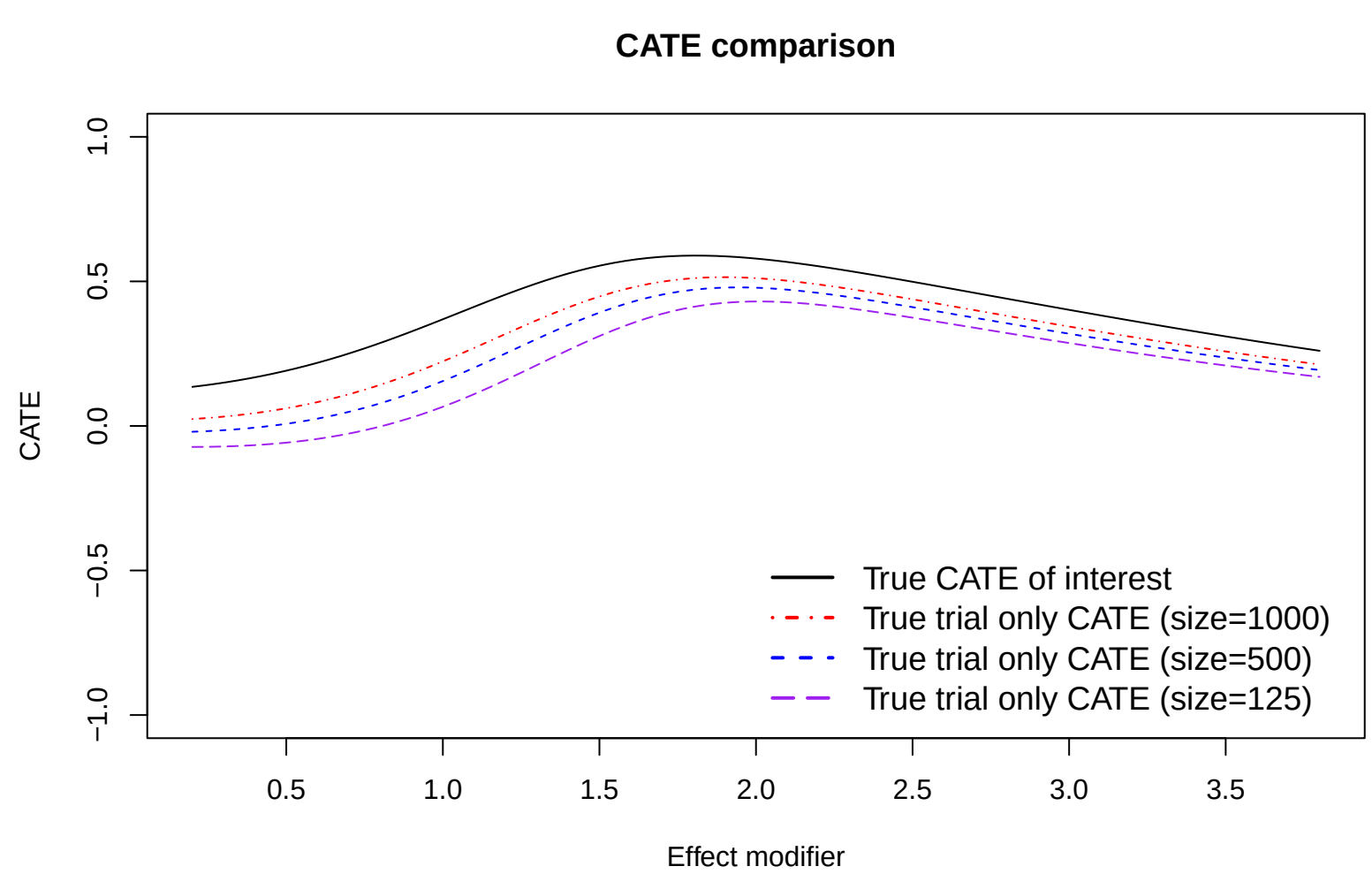

FIGURE D.4. A comparison of true CATE of interest among all trial-eligible individuals versus true CATE of trial only participants

Simulation results for this additional study is presented in Tables D.4 and D.5, and agree with the results in the main text. Table D.5 shows that results based on the trial only participants generally exhibited the largest amount of bias and mean squared error, as well as worst coverage of the 95% confidence intervals.

	Integrated |Bias|	Integrated RMSE	CP (%)
	$n_{s=1}$=125, n=2500		
Local linear regression	0.0201	0.3168	91
Linear regression (naive)	0.1177	0.3227	87
	$n_{s=1}$=500, n=2500		
Local linear regression	0.0196	0.1944	92
Linear regression (naive)	0.1175	0.2067	67
	$n_{s=1}$=1000, n=2500		
Local linear regression	0.0187	0.0863	89
Linear regression (naive)	0.1176	0.1361	40
	$n_{s=1}$=125, n=5000		
Local linear regression	0.0196	0.3165	89
Linear regression (naive)	0.1187	0.3212	87
	$n_{s=1}$=500, n=5000		
Local linear regression	0.0194	0.2084	92
Linear regression (naive)	0.1171	0.2157	69
	$n_{s=1}$=1000, n=5000		
Local linear regression	0.0179	0.1136	92
Linear regression (naive)	0.1173	0.1518	53

TABLE D.4. Supplementary simulation for binary outcomes: comparison of the proposed local linear and naive linear regression estimators.

	Integrated |Bias|	Integrated RMSE	Integrated CP
	$n_{s=1}$=125		
Local linear (trial only)	0.2036	0.2942	62
	$n_{s=1}$=500		
Local linear (trial only)	0.1772	0.2034	38
	$n_{s=1}$=1000		
Local linear (trial only)	0.1285	0.1436	40

TABLE D.5. Supplementary simulation for binary outcomes: results from local linear regression estimator based on trial only participants.

D.2.2 Continuous outcomes. We consider the same data generating mechanism for continuous outcomes as Subsection D.1, with the exception that outcomes for trial participants are generated according to the following Gaussian model: $Y \sim \mathrm{N}(\mu,1)$ where $\mu = 10+2A+X_1+X_2+X_3+(0.2X_2^2-0.1X_2^3+X_2X_3)A$.

There are two effect modifiers: (X_2,X_3), such that:

$$E(Y^{a=1}-Y^{a=0}|X)=2+0.2X_2^2-0.1X_2^3+X_2X_3.$$

As show in Figure D.5, difference between the true CATE in the trial eligible individuals differ greatly than the CATE in the trial participants. Indeed, due to the fact that $p(x_3|x_2,S=1) \neq p(x_3|x_2)$, we have that

$$E(Y^{a=1}-Y^{a=0}|X_2) \neq E(Y^{a=1}-Y^{a=0}|X_2,S=1)$$

and the difference is more prominent when the trial population size decreases (e.g., compare the CATE curve when the trial size is $n_{s=1}=125$ (in purple) to the curve when trial size is $n_{s=1}=1000$ (in red)).

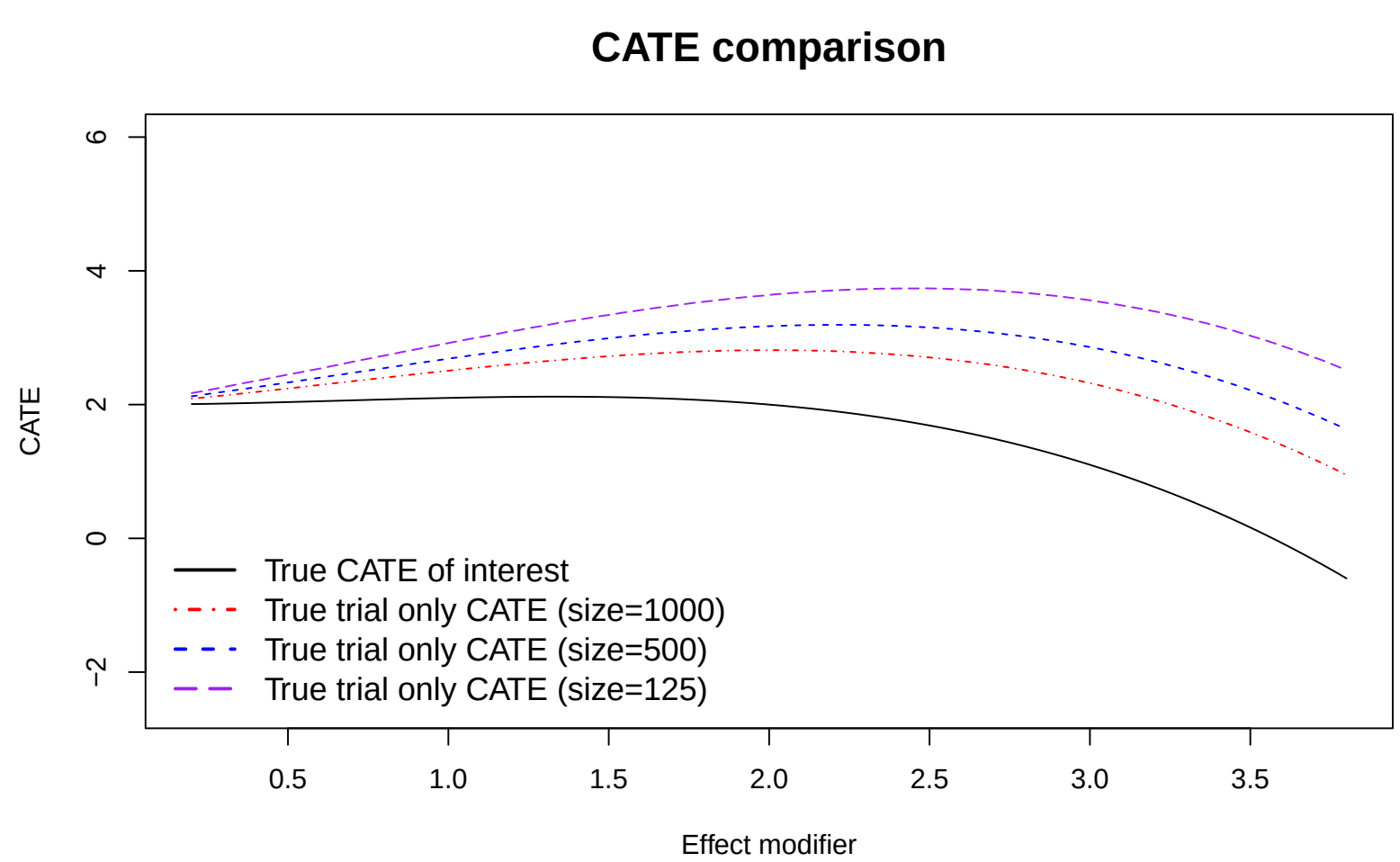

FIGURE D.5. A comparison of true CATE of interest among all trial-eligible individuals versus true CATE of trial only participants

Simulation results for this additional study is presented in Tables D.6 and D.7. Results in D.7 agree with the results presented in Table D.1.

Table D.6 shows that results based on the trial only participants generally exhibited the largest amount of bias and mean squared error, as well as worst coverage of the 95% confidence intervals.

	Integrated |Bias|	Integrated RMSE	Integrated CP
	$n_{s=1}$=125, n=2500		
Local linear regression	0.143	2.028	87
Linear regression (naive)	0.409	2.198	83
Oracle	0.218	2.272	90
	$n_{s=1}$=500, n=2500		
Local linear regression	0.069	0.728	90
Linear regression (naive)	0.369	0.770	68
Oracle	0.055	0.680	92
	$n_{s=1}$=1000, n=2500		
Local linear regression	0.072	0.348	88
Linear regression (naive)	0.368	0.477	50
Oracle	0.033	0.279	94
	$n_{s=1}$=125, n=5000		
Local linear regression	0.125	2.204	87
Linear regression (naive)	0.381	2.231	83
Oracle	0.166	2.334	91
	$n_{s=1}$=500, n=5000		
Local linear regression	0.071	0.906	89
Linear regression (naive)	0.369	0.880	70
Oracle	0.071	0.818	92
	$n_{s=1}$=1000, n=5000		
Local linear regression	0.064	0.498	90
Linear regression (naive)	0.369	0.579	57
Oracle	0.038	0.422	92

TABLE D.6. Supplementary simulation for continuous outcomes: comparison of the local linear, naive and oracle linear regression estimators.

	Integrated |Bias|	Integrated RMSE	Integrated CP
	$n_{s=1}$=125		
Local linear (trial only)	1.723	2.040	24
	$n_{s=1}$=500		
Local linear (trial only)	1.163	1.242	16
	$n_{s=1}$=1000		
Local linear (trial only)	0.755	0.794	14

TABLE D.7. Results from the local linear regression estimator based on trial only participants for continuous outcome.

E. Additional data application results

E.1 Trial only results. Figure E.6 shows the estimated CATE across the observed range of ejection fraction values using only trial data, stratified by myocardial infarction (MI) status. Both sets of plots show similar overall trends in CATE across ejection fraction and MI groups, but the trial-only estimates are more variable and exhibit sharper fluctuations in those who have no history of MI. In contrast, the data integration approach produces smoother and more stable curves.

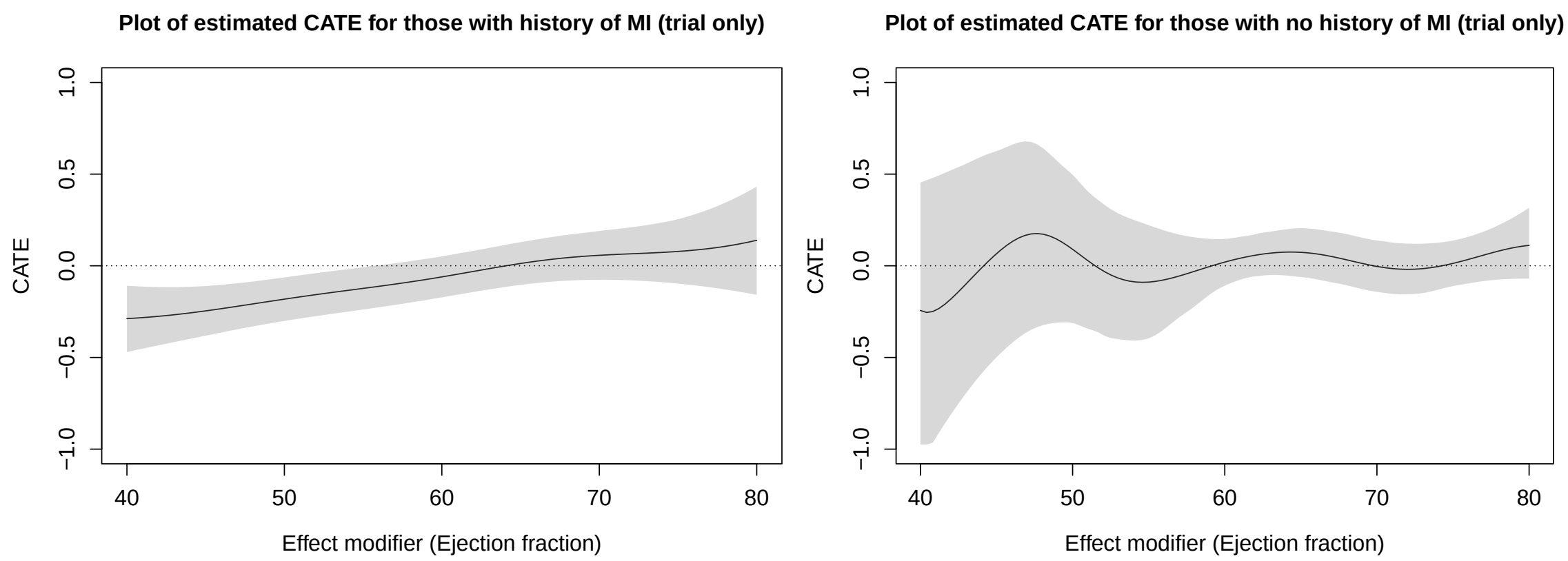

Figure E.6. Trial only estimated CATE stratified by history of myocardial infarction (MI) over the set of observed ejection fraction values from our proposed estimator.

References

Chernozhukov, V., Chetverikov, D., Demirer, M., Duflo, E., Hansen, C., Newey, W., and Robins, J. (2018). Double/debiased machine learning for treatment and structural parameters. *The Econometrics Journal*, 21:C1–C68.

Chernozhukov, V., Newey, W. K., and Syrgkanis, V. (2024). Conditional influence functions. *arXiv preprint arXiv:2412.18080*.

Dahabreh, I. J., Robertson, S. E., Tchetgen, E. J., Stuart, E. A., and Hernán, M. A. (2019). Generalizing causal inferences from individuals in randomized trials to all trial-eligible individuals. *Biometrics*, 75(2):685–694.

Kennedy, E. H. (2023). Towards optimal doubly robust estimation of heterogeneous causal effects. *Electronic Journal of Statistics*, 17(2):3008–3049.

Wen, L., Marcus, J. L., and Young, J. G. (2023). Intervention treatment distributions that depend on the observed treatment process and model double robustness in causal survival analysis. *Statistical methods in medical research*, 32(3):509–523.
